# Comparative Analysis of Membrane Vesicles from Three *Piscirickettsia salmonis* Isolates Reveals Differences in Vesicle Characteristics

**DOI:** 10.1371/journal.pone.0165099

**Published:** 2016-10-20

**Authors:** Julia I. Tandberg, Leidy X. Lagos, Petter Langlete, Eva Berger, Anne-Lise Rishovd, Norbert Roos, Deepa Varkey, Ian T. Paulsen, Hanne C. Winther-Larsen

**Affiliations:** 1 Center of Integrative Microbiology and Evolution, University of Oslo, Oslo, Norway; 2 Department of Pharmaceutical Biosciences, School of Pharmacy, University of Oslo, Oslo, Norway; 3 Department of Biosciences, University of Oslo, Oslo, Norway; 4 Department of Chemistry and Biomolecular Sciences, Macquarie University, Sydney, New South Wales, Australia; Hudson Institute, AUSTRALIA

## Abstract

Membrane vesicles (MVs) are spherical particles naturally released from the membrane of Gram-negative bacteria. Bacterial MV production is associated with a range of phenotypes including biofilm formation, horizontal gene transfer, toxin delivery, modulation of host immune responses and virulence. This study reports comparative profiling of MVs from bacterial strains isolated from three widely disperse geographical areas. Mass spectrometry identified 119, 159 and 142 proteins in MVs from three different strains of *Piscirickettsia salmonis* isolated from salmonids in Chile (LF-89), Norway (NVI 5692) and Canada (NVI 5892), respectively. MV comparison revealed several strain-specific differences related to higher virulence capability for LF-89 MVs, both *in vivo* and *in vitro*, and stronger similarities between the NVI 5692 and NVI 5892 MV proteome. The MVs were similar in size and appearance as analyzed by electron microscopy and dynamic light scattering. The MVs from all three strains were internalized by both commercial and primary immune cell cultures, which suggest a potential role of the MVs in the bacterium’s utilization of leukocytes. When MVs were injected into an adult zebrafish infection model, an upregulation of several pro-inflammatory genes were observed in spleen and kidney, indicating a modulating effect on the immune system. The present study is the first comparative analysis of *P*. *salmonis* derived MVs, highlighting strain-specific vesicle characteristics. The results further illustrate that the MV proteome from one bacterial strain is not representative of all bacterial strains within one species.

## Introduction

Membrane vesicles (MVs) are 50 to 250 nm spherical structures, enclosed by a single or double membrane, secreted from the surface of many Gram-negative bacteria during all stages of growth [1–3]. Proteomic and biochemical characterization has revealed that the vesicles contain a variety of bacterial components, including periplasmic and outer membrane proteins as well as lipopolysaccharides (LPS), DNA, RNA and cytoplasmic proteins [4–7]. Together they represent several aspects of the bacteria, but in a non-replicative form. MVs have also been reported to contain several important immunogenic factors, such as toxins [8], chaperons [9], and active enzymes [5]. The mechanisms of the MV formation and their biological role have yet to be clearly defined. However, bacterial MV secretion has been associated with several phenotypes including biofilm formation [10], bacterial survival [11], toxin delivery [12], cell-to-cell communication [13], and host-pathogen interactions [14]. MVs from infectious agents have also been found in both tissue and fluid samples from patients [15–17], indicating that the vesicle secretion plays an important role in the bacterial pathogenesis. The MV secretion is shown to be upregulated during stress and environmental changes [11]. These include treatment with membrane active antibiotics, nutrient depletion, temperature alteration and chemical exposure [18–21], alterations which the bacterium may encounter both in its natural environment and within a host. Alterations in MV production upon environmental changes can be exemplified by the human opportunistic pathogen *Pseudomonas aeruginosa*, which increases its secretion of MVs during treatment with gentamycin [18]. Similar observations have been done for *Shigella dysenteriae* serotype 1, which displayed a significantly higher concentration of the shiga toxin inside the MVs when treated with mitomycin C [19]. Pathogenic bacteria, in general, have the tendency to produce more MVs compared to their non-pathogenic counterparts, and for marine bacteria the vesicle production is reported to be important for survival [22–24]. Marine isolates of *Alteromonas* have been shown not only to persist, but also to grow in seawater media when supplemented with purified MVs from *Prochlorococcus*. In contrast, the control group displayed a reduced viability when grown in non-supplemented seawater media [24–25]. Furthermore, comparative MV protein profiling of two clinical isolates of *Acinetobacter baumannii* has revealed potential strain-specific links between vesicle content and virulence factors [26]. Taken together, it illustrates that the release of MVs may play an important role for bacterial survival and pathogenesis within a host.

In a host, isolated MVs have been shown to induce an immune response by activating the production of various cytokines, and have therefore been investigated and used as vaccines [4, 27]. However, modulating the immune response could also be beneficial for bacterial pathogens [28]. For a pathogen to successfully establish an infection, it needs to overcome the host’s initial immune defense [29]. The human pathogen *Moraxella catarrhalis* has been shown to utilize MV secretion in order to modify the B-cell response, to avoid direct contact with the host’s immune cells [30]. Furthermore, MVs form both *Helicobacter pylori*, *Pseudomonas aeruginosa* and *Neisseria gonorrhea* has been shown to upregulate the expression of nuclear factor NF-κB and the intracellular pattern recognition receptor NOD1 *in vitro*, promoting inflammation and pathology in infected hosts [31]. NF-κB is shown to be important for regulating the expression of several inflammatory and immune genes [32], while NOD1 has been described as a key pathogen recognition molecule (PRM) for the innate immune response [33]. Thus, the release of MVs interacting with the immune response could be beneficial for the pathogen in order to fight of the host’s defense system. Nonetheless, MV-based vaccines have successfully been used for epidemic control in Cuba, Norway, Brazil, and New Zealand against serogroup B meningococcal disease [34–37]. MVs used in vaccination of fish have also been reported to give good protection against *Edwardsiella tarda* in olive flounder *(Paralichthys olivaceus)* [38], *Flavobacterium psychrophilum* in rainbow trout (*Oncorhynchus mykiss*) [39], and *Francisella noatunensis* in zebrafish *(Danio rerio)* [40].

The Gram-negative intracellular bacterium *Piscirickettsia salmonis* is the etiologic agent of salmonid rickettsial septicaemia (SRS), a chronic and often fatal disease in salmonid and a variety of marine fish species [41–42]. *P*. *salmonis* was and characterized from Coho salmon (*Oncorhyncus kisutch*) in 1989 after a devastating epizootic in the Chilean aquaculture industry [41]. The bacteria has since then been recognized as an emerging problem as outbreaks of SRS has been reported across the world [43–45]. Strains of *P*. *salmonis* has been identified in salmon net-pens in Norway, Canada, Ireland and Scotland, but with a reduced virulence compared to the Chilean strains [46]. *P*. *salmonis* has been shown to infect, replicate and survive within macrophages as a part of its infection strategy. The infection process includes the formation of vacuoles within the host cells, enabling the bacterium to avoid the fish’s primary immune defense [44, 47–49]. The mechanisms behind *P*. *salmonis* ability to utilize macrophages are still poorly understood, but a Dot/Icm Type IV Secretion System homolog, has been identified within the genome of *P*. *salmonis*, and might be involved in the inhibition of phagosome-lysosome fusion during infection [50]. Furthermore, the heat shock protein ClpB and virulence factor BipA, proteins known to modulate the host cells defense mechanisms, has been reported to be expressed by the bacterium [51]. Nonetheless, the specific function of the Dot/Icm system, ClpB and BipA during SRS are still unknown. Thus, the mechanisms behind *P*. *salmonis* pathogenesis are poorly understood and further research is needed to characterize the bacterium.

MVs are of interest as they are considered to be important virulence factor and the secretion of MVs from *P*. *salmonis* was recently described for LF-89 [52]. As a geographic difference in virulence of SRS outbreaks have been reported [46], the present study focused on evaluating potential strain-specific differences in MV properties using three geographically disperse isolates of the bacterium including *P*. *salmonis* isolated from Norway (NVI 5692), Canada (NVI 5892) and Chile (LF-89). The identification and comparison of the proteins packed into MVs were analyzed to give new insight to the adaptation and virulence of *P*. *salmonis*. We show that intact MVs can be isolated from the three *P*. *salmonis* strains. Comparative MV profiling revealed several strain-specific factors, and in depth-analysis revealed that the vesicles contain a variety of proteins and that MVs may have a biological function both *in vivo* and *in vitro*.

## Material and Methods

### Bacterial Strains and growth conditions

Three isolates of *P*. *salmonis* were used for the characterization of MVs: LF-89 (type-strain ATCC VR 1361) isolated from Coho salmon (*Oncorhyncus kisutch*) in Chile [41], and NVI 5692 and NVI 5892 isolated from Atlantic salmon (*Salmon salar)* in Norway and Canada, respectively [53] (Kindly donated by Duncan J. Colquhoun, Norwegian University of Life Science). All three isolates were routinely grown at 20°C on Eugon Chocolate Agar (ECA), containing 30.4 g/L BD Bacto TM Eugon Broth (Becton, Dickinson and Company, Franklin lakes, NJ, USA), 15 g/L Agar Bacteriological (Thermo Fisher Scientific, Hudson, NH, USA) and 5% bovine blood (Håtunalab AB) [54] or in EBFC containing BD Bacto TM Eugon Broth supplemented with 2 mM FeCl3 (Sigma-Aldrich Co., St. Louis, MO, USA) and 1% Casamino Acids (BD) with agitation (100 rpm) for 7–10 days, depending on the isolate. The bacterial stocks used were frozen in autoclaved 10% skimmed milk (BD Difco) or in BD Bacto TM Eugon Broth supplemented with 20% glycerol (Sigma-Aldrich) and stored at—80°C.

### Purification and fluorescent labeling of MVs from *Piscirickettsia salmonis*

10 mL of exponential-growth phase cultures of each *P*. *salmonis* isolate was used to inoculate 200 mL of EBFC. The cells were grown at 20°C with agitation, and growth curves were measured by using optical density reading at 600 nm until the isolates reached late exponential-phase. MVs were isolated as described [40]. In short, the bacterial cells were removed by centrifugation (10 minutes, 15 000 g, 4°C), and the supernatant filtered sequentially through a 0.45- and 0.22 μm/pore filter in order to remove the remaining bacterial cells. The filtrate was then ultra-centrifuged sequentially at 125 000 g at 4°C for 2 hours and 125 000 g at 4°C for 30 minutes, to eliminate cell debris and aggregates. The MVs were resuspended in 100 μL 1x phosphate buffered saline (PBS) pH 7.2, and protein concentration determined by a Picodrop spectrophotometer (Picodrop Limited, UK). MV aliquots (10 μL) were spread onto ECA plates to check for sterility, and the remaining sample was stored at -80°C until use. A Zetasizer Nano ZS (Malvern instruments Ltd., UK) was used to conduct dynamic light scattering measurements, to determine the MVs size [55]. A velocity gradient centrifugation was preformed to evaluate the purity of the MV isolation, and each layer of the gradient investigated by transmission electron microscopy for quality control [7]. The labeling of MVs with fluorescein isothiocyanate (FITC; Sigma-Aldrich, USA) was done according to the method described [56], with some minor modifications. Vesicles were incubated for 1 hour at 25°C with 1 mg/mL isothiocyanate and pelleted at 25 900 rpm for 30 minutes. The FITC-labeled MVs were then washed three timed with 50 mM HEPES, resuspended in PBS and monitored for sterility and protein concentration as described above.

### Isolation of outer- and inner membranes using water lysis

10 mL of exponential-growth phase cultures of each *P*. *salmonis* isolate was used to inoculate 200 mL of EBFC. The cells were grown at 20°C with agitation until the isolates reached late exponential-phase. For the preparation of mixed membrane fractions the cell cultures were split into four sterile 50 mL Falcon tube and a water lysis protocol was used [57]. The mixed membranes were separated by a linear sucrose gradient of: 55%, 50%, 45%, 40%, 35% and 30% (w/w) sucrose in Tris-EDTA buffer. The mixed membrane samples were placed on top of each gradient and the samples ultra-centrifuged at 38 000 rpm at 4°C for 17 hours. The sucrose layers were carefully removed and the membrane fractions harvested, the inner membranes were at the 35–40% interface and the outer membranes at the 50–55% interface. The protein concentration was determined by a Picodrop spectrophotometer, and membrane aliquots (10 μL) were spread onto ECA plates to check for sterility. The remaining samples were stored at -80°C until use.

### SDS-PAGE

A standard SDS-PAGE procedure was used [58]. Briefly, 20 μg of membrane fractions and MVs isolated from LF-89, NVI 5692 and NVI 5892 was loaded onto a 12% (w/v) SDS polyacrylamide gel. The proteins separated through SDS-PAGE were stained with Coomassie Blue, and the image was acquired and evaluated using Gel doc^™^ XR+ with Image Lab^™^ software (Bio-Rad, Munich, Germany). Protein molecular weight standards were obtained from Bio-Rad.

### Electron Microscopy

For transmission electron microscopy carbon coated Formvar copper grids were placed on a drop of MV suspension for 5 minutes. The grids were then washed three times with PBS and the samples were fixed in 1% glutaraldehyde (Sigma-Aldrich) for 4 minutes. The samples were washed three times with PBS, two times with Milli-Q (MQ) water, stained for 20 seconds with 4% uranyl acetate (Sigma-Aldrich) in MQ water, washed once with MQ water and finally left on a solution of (9:1) methyl-cellulose (Sigma-Aldrich) with 4% uranyl acetate for 10 minutes on ice. The grids were then dried and viewed in a Philips CM200 transmission electron microscope and the images were acquired using the iTEM software (Olympus, PA, USA). For scanning electron microscopy a drop of bacterial suspension was placed on pre-coated poly-L-lysine (Sigma-Aldrich) coverslips (Thermo Scientific) and fixed overnight at 4°C with 2% glutaraldehyde in 0.1 M sodium cacodylate buffer pH 7.4. The coverslips were then washed twice in 0.1M sodium cacodylate buffer pH 7.4 for 10 minutes, and the samples dehydrated in a graded ethanol series for 10 minutes at 70%, 90%, 96% and 100% and for 15 minutes 4 times in 100% ethanol. Dehydrated samples were subsequently critical-point dried using carbon dioxide in a CPD 030 critical-point dryer (Bal-Tec, CA, USA), then mounted on stub with carbon-circles colloidal silver and sputter coated with a Cressington coating system 308R. The samples were viewed in a Hitachi S-4800 scanning electron microscopy, and images acquired using Scandium software (Olympus)

### Liquid chromatography-mass spectrometry

Three biological replicates of MVs harvested from *P*. *salmonis* LF-89, NVI 5692 and NVI 5892 were diluted to 40 μg of total protein in PBS and the samples were centrifuged at 16,000 g for 20 minutes at 4°C (Centrifuge 5415R, Eppendorf, Hamburg, Germany) and the supernatant discarded. Proteins were re-dissolved in 50 μL 6 M urea and 100 mM ammonium bicarbonate, pH 7.8. For reduction and alkylation of cysteines, 2.5 μL of 200 mM DTT in 100 mM Tris-HCl, pH 8 was added and the samples were incubated at 37°C for 1 hour followed by addition of 7.5 μL 200 mM iodoacetamide for 1 hour at room temperature in the dark. The alkylation reaction was quenched by adding 10 μL 200 mM DTT at 37°C for 1 hour. Subsequently, the proteins were digested with 10 μg trypsin (Promega, sequencing grade) overnight at 37°C. The digestion was stopped by adding 5 μL 50% formic acid and the generated peptides were purified using a ZipTip C18 (Millipore, Billerica, MA, USA) according to the manufacturer’s instructions, and dried using a Speed Vac concentrator (Concentrator Plus, Eppendorf, Hamburg, Germany). The tryptic peptides were analyzed using an Ultimate 3000 nano-UHPLC system connected to a Q Exactive mass spectrometer (Thermo Fisher Scientific, Bremen, Germany) equipped with a nano electrospray ion source (S1 File). The MVs from NVI 5692 and NVI 5892 were analyzed as routinely performed by the Australian Proteome Analysis Facility (APAF) and MVs from LF-89 by the Proteomic unit at the University of Oslo.

### Proteomic data analysis

Raw spectra files were converted into mgf format and processed using the global proteome machine (GPM) software with version 2.2.1 of X!Tandem algorithm [59] and a nonredundant output file was generated for protein identifications with log (e) values less than -1. Peptide identification was determined using a 0.8 Da fragment ion tolerance. Protein sequences extracted from the genome of LF-89 = ATCC VR-1361 [60] were used as the search database. A database of reversed sequences was searched to determine the false discovery rate at protein level. The three protein identification output files from each biological replicate of peptide samples were combined together to produce a single merged output file for each strains MV fraction. To ensure data quality, identified proteins were filtered based on two criteria: reproducible identification across three replicates and a total spectral count of >6, making the minimum number of peptides used to identify each protein an average value of 2 per replicate [61]. The subcellular location and functions for each of the identified MV proteins was predicted using PSORTb 3.0.2 [62] and their gene otology (GO) molecular function derived from The UniProt database [63]. The proteins were also subject to *in silico* analysis using VirulentPred, which predicts bacterial virulence proteins based on their sequences information [64].

### Flow analysis of MVs in fish cells

Zebrafish were anesthetized in tricaine methanesulfonate (MS-222, Sigma-Aldrich). Kidney and spleen were isolated as described [65]. Ten whole kidneys or spleens were pooled in 1 mL media; Leibovitz L-15 medium (Gibco) supplemented with 2% fetal bovine serum (FBS), penicillin (10 μg/mL) and streptomycin (10 μg/mL). Single-cell suspensions were generated by gentle teasing of the tissue on a 40 μm cell strainer with a plunger from a 1 mL syringe, collected in a 50 mL tube and rinsed twice with 1 mL media. The cells were cultivated in a concentration of 1x10^6^ cells/mL in 24 well plates. The SHK-1 *Salmo salar* macrophage-like cell line (passage 58) was maintained at 20°C in L-15 medium supplemented with 15% FBS in 25 cm^2^ flasks. For the microscopy imaging to observe cytopathic effect at different time points, 3 x10^5^ cells/mL were cultivated in μ-slide IV (Ibidi) with 20 μg/mL of MVs. Cells were analyzed with a Nikon inverted Microscope ECLIPSE TE300. The ability of kidney, spleen or SHK-1 cells to endocytose MVs *in vitro* was measured following methods described previously [66]. Briefly, 1 mL of cells (1x10^6^ cells) per sample was incubated for 1 hour at 20°C with 10 μg, 20 μg or 40 μg of FITC-MVs. After the incubation with MVs, the cells were washed three times with ice-cold phosphate buffered saline (PBS) and analyzed by flow cytometry using a Beckman Coulter (GaLLios). At least 10.000 events were collected for each sample. Data were analyzed using Kaluza software v.1.2 (Beckman Coulter) and macrophage/lymphocytes gated using Side scatter (SSC) (granularity) and Forward scatter (FSC) (size) parameters. Discrimination of aggregates from singlets was preformed using side scatter-W (SSC-W) versus side scatter (SSC) and Hoechst stains were used for the separation of dead and live cells. The fluorescence of the FITC conjugated MVs was measured before and after the addition of trypan blue (0,025% final concentration), to quench extracellular fluorescence. Incorporation of MVs-FITC was measured at 520 nm (FL1). The significant differences in percentage of MV uptake for each cell type was calculated using a Two-way ANOVA, Tukey`s multiple comparison test.

### Intraperitoneal injection of *Piscirickettsia salmonis* derived MVs in adult zebrafish

The biological effect of MVs *in vivo* were assessed by using 10–11 months old male and female Zebrafish *Danio rerio* wild type strain AB obtained from the model fish unit at the Norwegian University of Life Science. The fish were acclimatized to room temperature (20 ± 2°C) two weeks prior to the experimental setup. The fish were fed every morning with brine shrimp (Scanbur AS, Nittedal, Norway) and SDS 400 Scientific Fish Food (Scanbur AS) in the afternoon. Experimental groups of 20 fish were anesthetized by immersion in water containing 100 mg/mL tricaine methanesulfonate (MS-222, Sigma Aldrich) buffered with bicarbonate to pH 7–7.5. The fishwere injected intraperitoneally (i.p.) with 20 μL of PBS, 1x10^8^ colony forming units (CFU) of LF-89, NVI 5692, NVI 5892 or a total of 40 μg MVs in PBS isolated from LF-89, NVI 5692 and NVI 5892 respectively, by using a 27 g needle [40, 67]. After injection, the fish were immediately returned to recovery tanks and kept in separate 6-liters polycarbonate tanks (Pentair, USA), in which 50% of the water was manually changed daily. Fish that did not resume normal behavior after the injections were removed from the experiment and euthanized with an overdose of 250 mg/mL tricaine methanesulfonate. The water was provided by the model fish unit at the Norwegian University of Life Science and was supplemented with 0.55 g/L Instant Ocean sea salt, 0.053 g/L Sodium Bicarbonate and 0.015 g/L Calcium Chloride. The tanks were housed in a water-system with a controlled temperature (20°C) and with a cycle consisting of 14 hours of light and 10 hours of darkness. The fish were closely monitored, and the animal’s health recorded twice a day. Moribund or fish that clearly showed deviant behavior and clinical symptoms not consistent with good animal welfare (greatly reduced level of activity, response to environment and appetite), were euthanized as previously described. Water parameters were monitored every third day using commercial test kits (TetraTest kit): pH, NO_2_^-^, NO_3_^2-^, NH_3_/NH_4_^+^ and water hardness. All zebrafish experiment was approved by NARA (The Norwegian Animal Research Authority) and waste water decontaminated by chlorination and tested for sterility before disposal.

### RNA isolation and quantitative real-time PCR

For RNA isolation, three randomly chosen fish from each experimental group were sacrificed by an overdose of tricaine methanesulfonate (250 mg/mL) after 14 days, and kidney and spleen harvested. The organs were kept in RNAlater (Ambion) and stored at 4°C until further processing. The tissue was homogenized in 600 μL with buffer RLT (supplemented in RNeasy Mini Kit, QIAGEN) using a mortar and pestle (Sigma-Aldrich), followed by passing the lysate through a blunt 20 gauge needle fitted to a small 1 mL syringe (BD). Total RNA was extracted using the QIAGEN RNeasy kit according to the manufactures instructions, including a 15 minute on-column DNase treatment using an RNase-free DNase set (QIAGEN). The RNA was diluted in 30 μL RNase-free H2O (QIAGEN). RNA quantity and quality was measured with a Picodrop spectrophotometer. Reverse transcription reaction was performed by using High Capacity RNA to cDNA kit (Applied Biosystems). Quantitative real-time PCR (RT-qPCR) was carried out for each of the sampling points for a defined set of genes. These included major histocompatibility complex II (*MHC II*), cluster of differentiation 40 (*cd40*), interferon gamma (*ifnγ*), tumor necrosis factor alpha (*tnfα*), suppressors of cytokine signaling 3a and 3b (*socs3a* and *socs3b*), macrophage expressed gene 1 (*mpeg1*), nucleotide binding and oligomerization domain 1 and 2 (*nod1 a*nd *nod2*), and the six interleukins: *il-1β*, *il-6*, *il-8*, *il-10* and *il-12a*. QuantiTec bioinformatically validated primers were obtained from QIAGEN (Hilden, Germany) for most of the genes used; the remaining primers were obtained from Life Technologies Inc. (Carlsbad, CA, USA). Primers are listed in S1 Table. RT-qPCR was performed in triplicates using a Lightcycler^®^ 480 (Roche, Basel, Switzerland) as previously described [54]. 18S ribosomal RNA (*18S)* and Elongation factor-1 alpha (*ef-1α*) were used as reference genes for the normalization of the relative transcription levels of each gene, and the normalized immune response data of MV injected fish was standardized against the transcription levels of PBS injected fish for each time point. The significance of difference in relative gene expression levels between MV or bacterial challenges fish and PBS injected fish was calculated by a Student *t* test assuming unequal variance.

## Results and Discussion

### Isolation and phenotypic characterization of *Piscirickettsia salmonis* MVs

**Fig 1:**
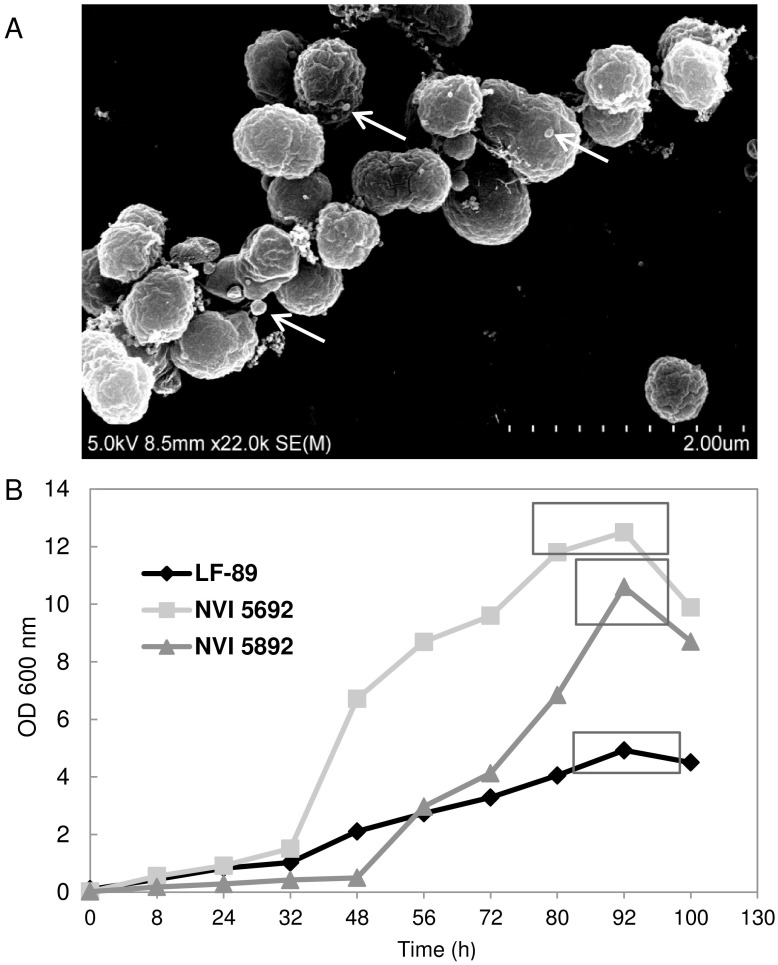
Identification and isolation of membrane vesicles isolated from *Piscirickettsia salmonis*. (A) Bacterial cultures of *P*. *salmonis* NVI 5692 grown in EBFC is viewed by scanning election microscopy. Arrows indicate MVs secreted from the bacterial cells. (B) Growth curves of *P*. *salmonis* in EBFC medium, square show time point for isolation of MV (n = 3).

The *P*. *salmonis* derived MVs were observed on the surface of bacterial cells when exanimated by scanning electron microscopy, demonstrating that vesicles may bud off from the bacterial membrane during growth in liquid medium (Fig 1A). All three strains of *P*. *salmonis*, LF-89, NVI 5692 and NVI 5892 produced small spherical MVs when grown to late exponential-phase in EBFC medium. *In vitro* growth has previously been obtained for *P*. *salmonis* up to an optical density of OD_620_ = 1.8 in AUSTRAL-SRS medium [68] and OD_600_ = 2 in BM1 [69]. For NVI 5692 and NVI 5892 the late exponential to stationary growth-phase is reached at an optical density of OD_60 0_ = 10–12 with a measured CFU ~5x10^9^ in EBFC (Fig 1B). LF-89 expressed a reduced growth pattern in comparison to the other strains, reaching its late exponential to stationary growth-phase at an optical density of OD_600_ = 4–5 with CFU measured to ~2x10^9^ (Fig 1B). Thus, the EBFC medium enhances the optimal growth of the three *P*. *salmonis* isolates up to six fold from previously published media. The three strains do, however express a divergent growth pattern in EBFC reaching different optical densities within the same period, which could potentially affect the MV production, lipid content and protein composition. As NVI 5692 and NVI 5892 reach their exponential-phase between OD_600_ = 10–12, the two strains will have a higher cell density prior to the isolation compared to LF-89, thus the bacterial cultures were diluted to an equal cell number before harvesting the vesicles [70]. Alternatively, MVs could be harvested from NVI 5692 and NVI 5892 at an optical density of OD_600_ = 4–5, but the cultures would then not have been in late exponential-phase in contrast to LF-89. MVs isolated from *P*. *aeruginosa* grown in both cultures has been reported to display differences in both lipid and protein composition when harvested from exponential and stationary phase. These data indicate that the MV properties could be growth phase dependent [71]. Furthermore, as the different stages of bacterial growth has been reported to affect protein expression in general [72–73], harvesting MVs from cultures in an equal growth phase is preferable over a variation in cell density.

**Fig 2:**
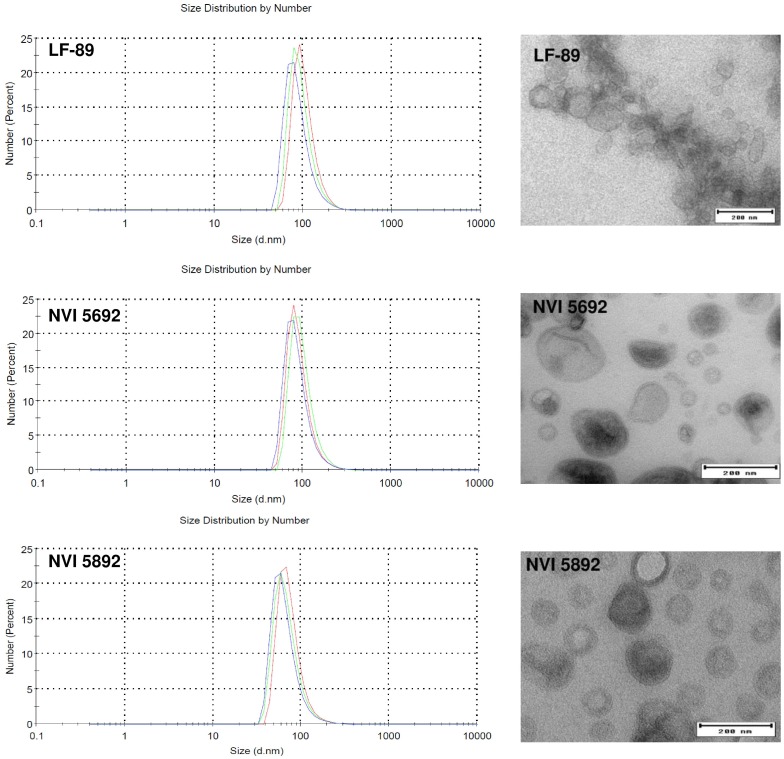
Size distribution and imaging analysis of *Piscirickettsia salmonis* membrane vesicles. Vesicle size and range analyzed by dynamic light scattering (left panels) (n = 3) and electron transmission microscopy imaging (right panels) of MVs isolated from LF-89, NVI 5692 and NVI 5892. Bar size, 200 nm.

Examination of the MVs isolated from EBFC medium by transmission electron microscopy and dynamic light scattering revealed a phenotypical similarity between the three strains of *P*. *salmonis*, analogues in both size and distribution (Fig 2). MVs from all three strains were also dominated by double membrane vesicles (S1 Fig), indicating that the MVs contain both an plasma and outer membrane, similar to what has been described for other Gram-negative bacteria [3]. Vesicles isolated from LF-89 had a higher tendency to form clusters of MVs compared to NVI 5692 and NVI 5892, however, some collections of vesicles were observed for all strains. Both image analysis using the iTEM software and Dynamic light scattering was used to determine the size distribution for the MVs between the three stains of *P*. *salmonis*. All strains were shown to have MVs ranging from 10–220 nm in size, with an average between 80–100 nm (Fig 2), similar to what has been reported for other bacterial species [5, 74–75]. The isolated vesicles from all three strains of *P*. *salmonis* were also compared to their outer membrane fractions by SDS-PAGE and coomassie blue staining (S2 Fig). Although not directly quantitative, coomassie blue staining is useful to examine differences in protein composition between samples. For all three strains of *P*. *salmonis* the vesicles resembled, but were not identical to the membrane fractions. This indicates that the vesicles are purified fractions and not membranes from lysed bacterium; however, the presence of non-MV associated material cannot be completely excluded.

### Identification of MV proteins from *Piscirickettsia salmonis* and their predicted subcellular distribution

**Fig 3:**
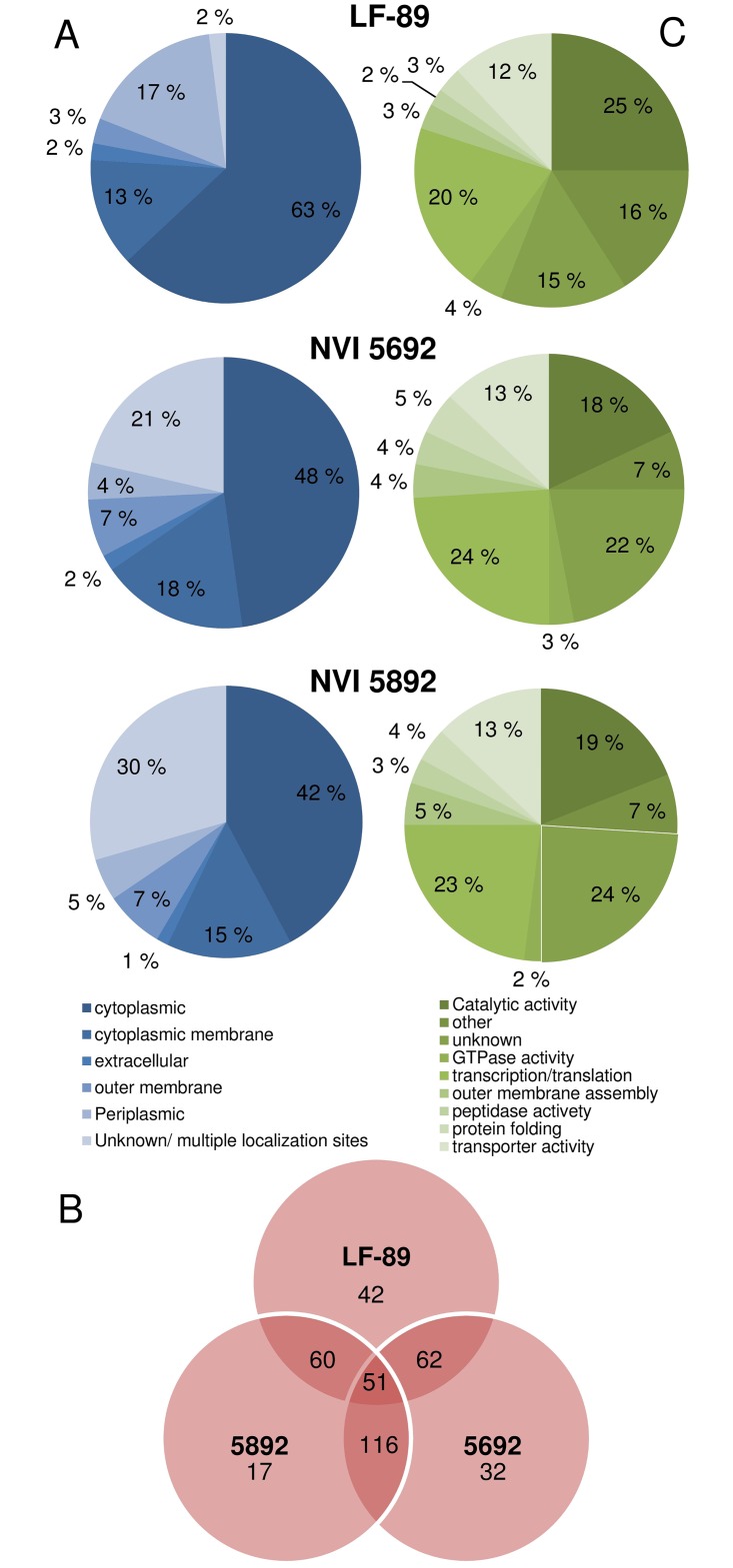
Proteomic characterizations of *Piscirickettsia salmonis* membrane vesicles. The identified proteins in the MVs were grouped into families according to their (A) predicted subcellular localization and (C) putative function. (B) Venn diagram comparing MV proteins from three different strains of *P*. *salmonis*.

The total number of proteins identified from liquid chromatography-mass spectrometry (MS) of *P*. *salmonis* derived MVs identified 119 (FDR 0.18%) different proteins in vesicles isolated from LF-89, 159 (FDR 0.16%) from NVI 5692 and 142 (FDR 0.16%) from NVI 5892 (S2–S4 Tables). However, as the genomes of NVI 5692 and NVI 5892 have not been sequenced, the identified proteins by MS are based on the genome of LF-89 for all three strains, limiting the protein identification. The PSORTb 3.0.2 identified the potential subcellular localization of 98%, 79% and 70% of the *P*. *salmonis* MV proteins identified for LF-89, NVI 5692 and NVI 5892, respectively. The majority of the identified proteins (~ 60%) in MVs isolated from *P*. *salmonis* were predicted to be cytoplasmic proteins (Fig 3A). The high number of cytoplasmic proteins is most likely do to the presences of a double membrane in the majority of the MVs (S1 Fig). As the formation of double membrane vesicles are characterized by a disruption of both the plasma and outer membrane, high amounts of cytoplasmic proteins have been shown to be packed into the MVs [3]. We cannot, however, fully exclude the presence of cytoplasmic contaminants in the samples, which could contribute to a higher number of proteins been identified as cytoplasmic. The presence of several cytoplasmic proteins have, nonetheless, been identified in proteomic studies of several bacterial derived MVs [5, 9, 76–77], and it has been suggested that some of these proteins could be sorted into the vesicles during the MV formation [2, 78–79]. This may implicate that the MV production is specific and not a random event, allowing for selective incorporation of proteins into the vesicles. Compared to the hypothetical proteome of *P*. *salmonis* LF-89 (ATCC VR 1361) (S3 Fig), the cytoplasmic proteins were downregulated in the vesicles, while outer membrane proteins were enriched. An enrichment of outer membrane proteins in bacterial derived vesicles has been described for several other Gram-negative bacteria including *Vibrio cholerae* [80], *Neisseria meningitides* [81] and *Mycobacterium tuberculosis* [77]. An escalation of outer membrane proteins in the *P*. *salmonis* derived MVs, may therefore play an important role in the biological function of the vesicles, as membrane proteins often function as an interface between the pathogen and its host. An in-depth analysis of the MV proteins revealed a strain-specific difference, were the highest similarity were observed between the vesicles from NVI 5692 and NVI 5892 (Fig 3B). Strain-specific variations of MV content has been reported for *Haemophilus influenza*, revealing that certain outer membrane proteins were enriched or excluded in MVs from different isolates [82]. A variation between the three isolates is therefore not unique for *P*. *salmonis*, however, why the LF-89 derived MVs differentiates from the Norwegian and Canadian strain has yet to be revealed.

### Functional classification of proteins identified in *Piscirickettsia salmonis* MVs

The identified MV proteins were further categorized based on their predicted functions, where limited differences were identified between the MVs from the three stains of *P*. *salmonis*. Both proteins involved in translation/transcription and catalytic activity were abundant in all the samples (Fig 3C). Proteomic profiling during different stages of bacterial growth has shown that proteins involved in DNA and RNA synthesis are upregulated during the log-phase [72–73, 83]. A study of the *Acinetobacter baumannii* proteome has shown that several translation-related proteins are upregulated during both exponential and early stationary phase. These include 50S ribosomal protein L3, L5, L6, 30S ribosomal protein S2, S8 and elongation factor G and Tu [84]. Therefore, proteins involved in transcription and translation may naturally be packed into the vesicles, as these are upregulated by the bacterium during early growth stages. A differentiation in protein levels has been observed for MVs harvested from *Salmonella enterica* grown in different medium. MVs isolated from *S*. *enterica* grown in LB medium were reported to have higher number of translation/transcription proteins compared to *S*. *enterica* grown in acidic MgM media. The MgM cultures on the other hand, had a higher abundance of proteins involved in transporter activity [85]. Thus, the bacterial growth conditions could have an impact on the vesicles protein composition. However, growth phase dependent packing of *P*. *salmonis* MVs was not performed in the present work and will be interesting to investigate in future studies.

In addition to a high abundance of translation/transcription proteins in the *P*. *salmonis* MVs, approximately one fourth of all the proteins were assigned a catalytic activity. The identification of catalytic proteins in MVs is reported in several species [4, 14, 86] and the packing of active enzymes into MVs is proposed to have an important role in virulence. This can be exemplified by studies of *Pseudomonas aeruginosa* derived MVs, which, has been shown to contain active chromosomally encoded β-lactamase [87]. β-lactamases are enzymes that deactivate β-lactam antibiotics like penicillin and cephamycins by interruption of the β-lactam ring and thus its activity, providing bacterial resistance against antibiotic treatment [88]. Furthermore, *P*. *aeruginosa* has been reported to upregulate its production of MVs in the presence of antibiotics [86]. Therefore, the presence of enzymatic proteins within MVs may provide increased survival and resistance during infections. Proteins displaying catalytic activity have also been reported in non-virulent bacteria, including *Bacteroides fragilis* and *Bacteroides thetaiotaomicron*, members of the human microbiota. Both *B*. *fragilis* and *B*. *theaiotaomicron* derived MVs were reported to display sugar-hydrolyzing activity [89]. The catalytic activities of the *P*. *salmonis* derived MVs were not determined in this study, but based on the bioinformatics analysis the vesicles harbor a variety of enzymes.

### Identification of strain-specific MV proteins and their relation to virulence and adaptation

The full protein content of all three strains of *P*. *salmonis* derived MVs is listed in the S2–S4 Tables, while the 20 most abundant proteins identified by mass spectrometry analysis are shown in Table 1. As the proteomic analysis of the MVs was performed on a collection of vesicles, rather than a single MV, the 20 most abundant proteins are most likely presents in multiple MVs, due to the high number of total mass spectra assigned to the individual proteins. Of the 20 most abundant proteins, six proteins are common for all the strains, while ten proteins overlap between NVI 5692 and NVI 5892. These data illustrate a higher similarity between the Norwegian and Canadian strains compared to the Chilean. When taking the total proteomic profile of the strains-specific vesicles into account, similar findings were identified. 42 proteins were identified only in the vesicles derived from LF-89, in contrast to 32 in NVI 5692 and 17 in NVI 5892 (Fig 3B). To which degree these individual differences in protein content affects the vesicles biological role is not known, but they might contribute to a differentiation in virulence and adaptation for the three strains of *P*. *salmonis*. NVI 5692 and NVI 5892 are both isolated from Atlantic salmon from the northern Atlantic Ocean in Norway and Canada respectively, while LF-89 isolated from Coho salmon form the South Pacific Ocean in the south of Chile. Strains of *P*. *salmonis* found in the south of Chile, including LF-89, are reported to cause a high mortality rate in salmonids, with an accumulating mortality reaching almost 90% [90–91]. Outbreaks of *P*. *salmonis* in Norway and Canada have been, in contrast to Chile, less severe. Out of 14 fish farms in Norway affected during 1988–1992, only 35% of the fish displayed multiple symptoms of SRS. The mortality rate has also been, as in Canada, subsequently lower, ranging from 2–30% [92–93]. The reason for the higher severity of *P*. *salmonis* outbreaks in Chile compared to other graphical areas is yet to be revealed. Different strains of *P*. *salmonis* have been shown to be closely related independently of their geographical distribution, as 16S and intergenic spacer ITS-1 sequencing of different isolates has shown that strains from both Chile, Norway and Canada has a high phylogenetic similarity [94–96]. Other factors, including environmental and geographical variations, might therefore contribute to a variation in virulence among the different strains of *P*. *salmonis*.

**Table 1:** Top 20 proteins most commonly identified by label-free shotgun proteomics in *Piscirickettsia salmonis* membrane vesicles.

LF-89		NVI 5692		NVI 592	
Identified protein	Total number of spectra	Identified protein	Total number of spectra	Identified protein	Total number of spectra
Outer membrane family protein	81	Putative uncharacterized protein*	144	Putative uncharacterized protein*	171
DNA-directed RNA polymerase subunit beta	79	Putative uncharacterized protein**	136	Peptidyl-prolyl cis-trans isomerase**	140
Bacterial DNA-binding family protein*	72	Prolyl oligopeptidase family protein**	133	Outer membrane beta-barrel domain protein**	104
Chaperone protein DnaK*	63	Type I secretion outer membrane TolC family protein**	89	Outer membrane protein assembly factor BamA**	89
60kDa chaperonin GroEL	58	SH3 domain of the SH3b1 type family protein*	86	Prolyl oligopeptidase family protein**	83
30s ribosomal protein S1*	44	Conjugal transfer/type IV secretion DotA/TraY family protein**	81	Type I secretion outer membrane, TolC family protein**	82
SH3 domain of the SH3b1 type family protein*	44	Outer membrane beta-barrel domain protein**	81	Outer membrane family protein	79
ATP synthase subunit beta	40	Outer membrane protein assembly factor BamA**	72	Chaperone protein DnaK*	74
Succinyl-CoA synthetase subunit beta	37	30s ribosomal protein S1*	69	SH3 domain of the SH3b1 type family protein*	72
Adenylosuccinate synthetase	35	Peptidyl-prolyl cis-trans isomerase**	65	Conjugal transfer family protein**	71
50S ribosomal protein L2*	35	Bacterial DNA-binding family protein*	65	OmpA family protein	68
Pyruvate dehydrogenase E1 component	35	50S ribosomal protein L2*	64	Outer membrane protein assembly factor BamD**	64
Translation elongation factor Tu	32	Outer membrane protein assembly factor BamD**	63	Bacterial DNA-binding family protein*	62
ATP synthase subunit alpha	31	Chaperone protein HtpG	60	Conjugal transfer/type IV secretion DotA/TraY family protein**	61
30S ribosomal protein S10	31	Conjugal transfer family protein**	60	Glycerophosphoryl diester phosphodiesterase family protein**	57
Acetyl-CoA carboxylase, biotin carboxylase subunit	30	NAD-specific glutamate dehydrogenase	59	50S ribosomal protein L2*	57
GTP-binding protein TypA/BipA	30	ATP synthase subunit alpha	57	30s ribosomal protein S1*	55
Adenylosuccinate lyase	30	Chaperone protein DnaK *	57	Putative uncharacterized protein	52
Putative uncharacterized protein*	29	Glycerophosphoryl diester phosphodiesterase family protein**	56	ostA-like family protein	48
Glutamine synthetase	28	Succinyl-CoA synthetase subunit beta	50	DSBA-like thioredoxin domain protein	46

*Proteins identified in MVs from all three strains of *P*. *salmonis*

**Proteins identified in MVs from *P*. *salmonis* strains NVI 5692 and NVI 5892

Nonetheless, the three strains have several proteins in common, and many of these proteins are highly represented in the vesicles of all three isolates. This indicates that MVs can have similar functions although isolated from three geographically disperse strains of *P*. *salmonis*. To evaluate the potential virulence of the *P*. *salmonis* derived MVs, the vesicles were subjected to *in silico* analysis using VirulentPred, to predict putative virulence factors [64]. Based on the VirulentPred analysis, almost 50% of the MV proteins were predicted to be associated with virulence in all three strains (results not shown). Some of these proteins were in addition among the most commonly identified protein by the proteomic analysis, which includes TolC, GroEL and DnaK (Table 1). TolC is involved in multidrug resistance and has previously been described as an virulence factor in the human pathogen *Francisella tularensis* [97]. Deletion of the TolC orthologue in *F*. *tularensis* did exhibit a significant reduction of virulence in mice, suggesting that TolC is involved in the bacterial pathogenesis of *F*. *tularensis* [97]. TolC has also been reported to be important for environmental adaptation, protein secretion and drug resistance in several Gram-negative bacteria [98]. The identification of TolC in *P*. *salmonis* derived MVs may reflect their presence in the bacterial membrane, although it is not known if the proteins play an active role in the bacterial vesicles. While TolC is identified as the top six most abundant proteins in NVI 5692 and NVI 5892, it does not reach the top 20 list in the LF-89 strain (Table 1).

Certain proteins do not need to be active to have a biological role. This can be exemplified for chaperone proteins, including GroEL and DnaK, which initially prevents protein aggregation by either refolding or degrading misfolded proteins [99]. Both of these proteins has been shown to be highly immunogenic independently of their function [100], and reported to induce the expression and release of the pro-inflammatory cytokines IL-6 and tumor necrosis factor alpha (TNFα) in human monocytes, both individually and in combination [101–102]. Treatment of HUVEC cells with *Escherichia coli* derived GroEL and DnaK has further been shown to upregulate the release of intercellular adhesion molecule-1 (*ICAM-1*), and vascular cell adhesion molecule-1 (*VCAM-1*), important for the recruiting of leucocytes, in addition to IL-6 in a dose dependent manner [101]. Thus, the high abundance of GroEL and DnaK in *P*. *salmonis* derived MVs might contribute to an increased immunogenic effect of the vesicles.

### Dose-dependent internalization of MVs by *in vitro* cell cultures

**Fig 4:**
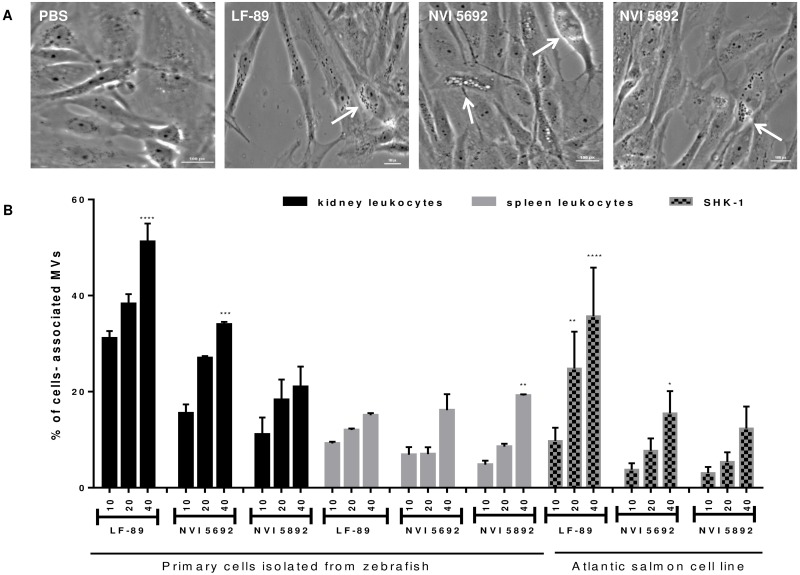
Internalization and effect of membrane vesicles isolated from *Piscirickettsia salmonis* in fish cells. (A) Cytopathic effect of 20 μg/mL MVs in SHK-1 cells. The cytopathic effect is characterized by the production of rounded vacuoles (arrow). Bar size, 100 μm. (B) The effect of three different MV concentrations (10, 20 and 40 μg/mL) on internalization in SHK-1 cells and kidney and spleen primary leukocytes isolated from adult zebrafish assessed by flow cytometry (n = 3). Results are presented as mean ± SD. Asterisks indicate statistical significances between the different concentrations of MVs within each cell type (Two-way ANOVA, Tukey`s multiple comparison test). P value: **** < 0.0001; *** < 0.001; ** < 0.01; * < 0.1.

To investigate the biological role of the *P*. *salmonis* derived MVs, the vesicles interactions with both commercial and primary cell cultures were assessed by microscopic examination and flow cytometry. As the appearance of a cytopathic effect (CPE) has previously been used to evaluate the susceptibility of cell lines to *P*. *salmonis* [42, 103], the CPE after exposure to MVs was evaluated using a salmon head-kidney cell line (SHK-1) (Fig 4A). A 20 μg/mL concentration of *P*. *salmonis* derived vesicles was added to SHK-1 cultures, and the CPE was observed after 48 hours by the formation of round vacuoles within the cell (Fig 4A). *P*. *salmonis* have previously been reported to infect SHK-1 cells [104], and the CPE induced by vesicles isolated from *P*. *salmonis* might therefore indicate a virulent effect of the MVs. Cellular damage caused by bacterial vesicles has been reported in a variety of cell lines as exemplified by RAW264.7, THP-1, and HL60 cells treated with MVs from *Acinetobacter baumanii*, *Aggregatibacter actinomycetemcomitans* and *Actinobacillus actinomycetem- comitans*, respectively [26, 76, 78]. The secretion of MVs has also been observed from the fish pathogen *Francisella noatunensis* subsp *noatunensis* during infections in primary cod leukocytes and within zebrafish embryos [40, 105].

The interaction between *P*. *salmonis* derived MVs and cultured cells were further evaluated using flow cytometric analysis in combination with FITC-labeled vesicles. A concentration of 10 μg/mL of FITC conjugated MVs from all three strains of *P*. *salmonis* were shown to be internalized by both kidney and spleen primary leukocytes isolated from adult zebrafish (S4 Fig). To investigate the dose-response in the incorporation of MVs two additional doses of FITC conjugated MVs (20 and 40 μg/mL) from all three bacterium were incubated with primary culture or SHK-1, and the incorporation analyzed by flow cytometry. Increasing concentrations of vesicles resulted in a linear enrichment of MVs association with zebrafish primary cells and in SHK-1 cells (Fig 4B). Vesicles isolated from LF-89 were shown to display a significantly higher degree of association with primary zebrafish kidney leukocytes compared to SHK-1 cells and primary zebrafish spleen leukocytes (p<0.001). NVI 5692 and NVI 5892 derived MVs displayed a similar internalization in all three cell types (Fig 4B).

A dose-dependent uptake of MVs has previously been described for *Brucella abortus* [56]. Moreover, *B*. *abortus* derived vesicles has been reported to modulate the innate immune response in human epithelial and monocytes cells, as well as increasing the adherence and internalization of *B*. *abortus in vitro* [56]. In general, the cellular process for internalization of MVs is not known, but studies with *B*. *abortus* and *H*. *pylori* has shown a potential uptake of MVs by the clathrin-mediated endocytosis, the main pathway for receptor-mediated endocytosis in most eukaryotic cells [56, 106–107]. A similar infection strategy has been suggested for *P*. *salmonis*, indicating that the utilization of macrophages is dependent upon the interaction with host-cell clathrin and actin [108]. The capability to exploit host cells for survival by manipulating cellular processes by protein secretion and specific effectors has been described for a range of pathogens [109]. E.g. membrane vesicles isolated from *Legionella pneumophila* inhibit the fusion of phagosomes with lysosomes in primary mouse macrophages [110]. Therefore, *P*. *salmonis* could potentially utilize the MV secretion as a survival strategy to replicate within macrophages. Both the primary and commercial cell lines revealed a higher association with LF-89 derived MVs in contrast to NVI 5692 and NVI 5892 in this study. This suggests that MVs might promote bacterial survival within macrophages, which could explain the higher virulence reported for the Chilean strain. Thus, bacterial release of MVs within the host may contribute to the utilization of host cells and modulations of the immune system during an infection. To which degree MVs are released during SRS outbreaks has yet to be investigated but was recently shown in CHSE-cells [52].

### Studies of *Piscirickettsia salmonis* derived vesicles in adult zebrafish

**Fig 5:**
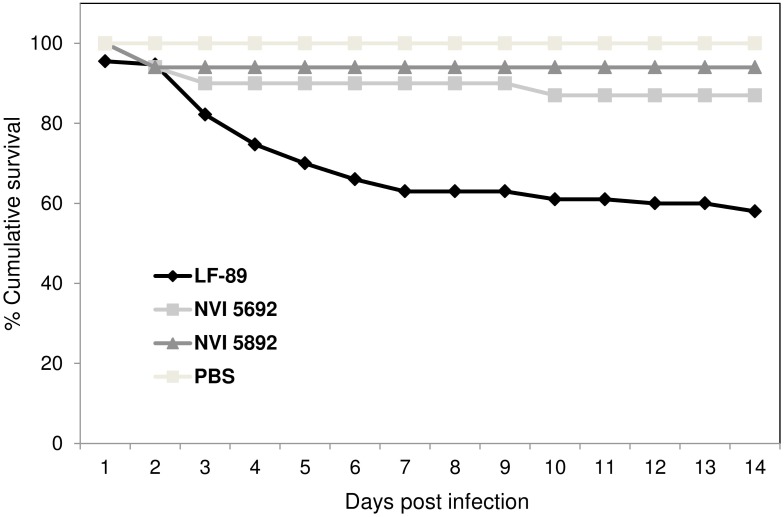
Adult zebrafish challenged with membrane vesicles isolated from *Piscirickettsia salmonis*. Cumulative survival of adult zebrafish injected with 40 μg of MVs isolated from the three different strains of *P*. *salmonis* (LF-89, NVI 5692 and NVI 5892) or PBS (n = 20).

**Fig 6:**
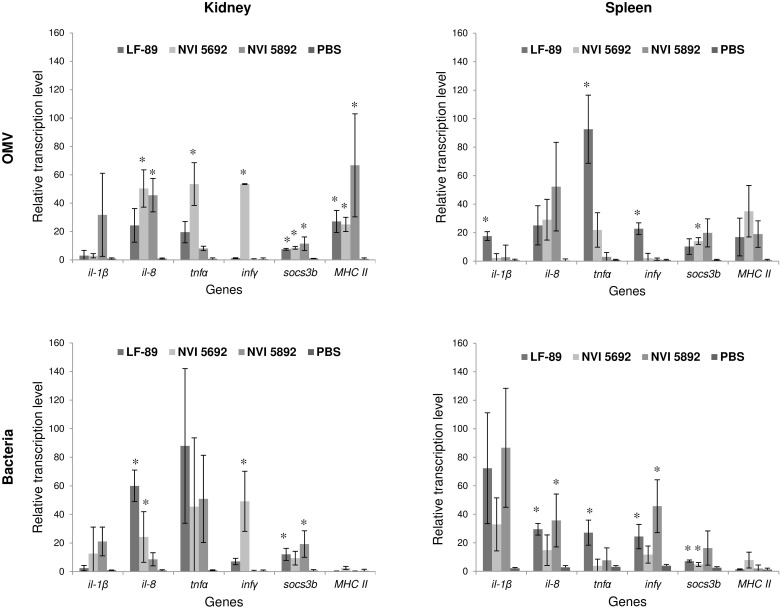
Immune gene transcription of adult zebrafish challenged with *P*. *salmonis* and isolated membrane vesicles analyzed by RT-qPCR. Immune gene expression of kidney and spleen, isolated 14 days post injection with either 40 μg MVs isolated from three different strains of *P*. *salmonis* or 1x10^7^ CFU of the same bacteria strains (LF-89, NVI 5692 and NVI 5892). Results are presented as mean +/- SD. Asterisk indicate significantly upregulated genes compared to the PBS control p<0.05, two tailed unpaired Student’s t-test (n = 3).

To study the potential effect of MVs *in vivo*, adult zebrafish were injected with MVs from the three strains of *P*. *salmonis*. In recent years, zebrafish has proven to be a unique model for the study of leukocytes subset, immune cell migration and host-pathogen interaction [111]. The effects of MVs have previously only been described for *F*. *noatunensis* in a zebrafish model [40], with no observed cytotoxic effects. For zebrafish injected with 40 μg MVs from NVI 5692 and NVI 5892, no behavioral alterations were observed over two weeks compared to the PBS control group (Fig 5). Interestingly, fish injected with MVs from LF-89 presented a reduction in activity and approximately three days post injection mortalities were detected. A rapid decrease in accumulative survival up to 50% were registered for the LF-89 MV group during the first seven days, indicating an initial acute phase, which stabilized by day 9 (Fig 5). In the NVI 5692 and NVI 5892 MV group, less than 10% mortalities were registered, most likely due to complications from the injections, as they occurred shortly after the procedure. Kidney and spleen samples were harvested two weeks post-injection to evaluate the MVs immunogenic effect in the fish. Of the genes analyzed no significant up or down regulation were detected for *il-*6, *il-10*, *il-12a*, *socs3a*, *mpeg1*, *cd40*, *nod1* and *nod2*. This could mean that the MVs do not affect the selected genes, or that it occurs at an earlier time point. An increased expression of immune genes was, however observed for *il-1β*, *il-8*, *tnfα*, *infγ*, *socs3b and MHC II* in all three groups injected with *P*. *salmonis* derived MVs (Fig 6). The gene expression profile for the fish challenged with MVs were in most cases similar to the ones challenged with live bacteria, indicating that the vesicles mimic their mother cells. Several pathogens, including the intracellular ones, modify the suppressor of cytokine signaling (Socs) to inhibit the host’s ability to clear an infection [112]. Thus, the effect of MVs on the *socs3b* gene expression was investigated, showing a significant increased expression. Such alterations of the cytokine secretion is a common modification initiated by several intracellular pathogens, enabling prolonged utilization of macrophages, by downregulating the host’s defense mechanisms [113]. To what degree the *P*. *salmonis* derived MVs can modulate the cytokine expression has not previously been explored. However, as *socs3b* in combination with several other immune related genes were upregulate after exposure to MVs, the *P*. *salmonis* derived vesicles might display immunogenic abilities. However, further studies including sampling at earlier time points are needed to fully evaluate the potential immunogenic effect of the vesicles.

These immunogenic abilities are, nonetheless, partly the reason why MVs over the last decades have been explored and successfully used as vaccine components [1, 36]. Thus, several pro-inflammatory genes were investigated and shown to be significantly upregulated in zebrafish injected with the *P*. *salmonis* derived vesicles, including *il-1β*, *il-8*, *tnfα* and *infγ*. The inflammatory cascade of an infection begins with receptors involved in the binding and uptake of infectious agents and their products by cells of the innate immune system. This is then followed by the production of pro-inflammatory cytokines, such as TNFα, IL-1, IL-8 and IFNγ [114]. In the present work, we show that *P*. *salmonis* derived MVs are able to upregulated genes involved in an inflammatory response, as well as genes related to antigen representing cells, (*MHC II*) in an adult zebrafish model. Interestingly, the *MHC II* genes were significantly upregulated in fish injected with MVs compared to fish injected with live bacteria (p<0.05). However, as *P*. *salmonis* is an intracellular bacteria utilizing macrophages as a part of its infection strategy, a low *MHC II* expression might be expected [115]. On the other hand, the MVs do not replicate within a host, and might therefore be taken up and degraded by antigen representing cells, increasing the *MHC II* expression, which also makes them interesting as potential vaccine components. Several of the genes investigated have been reported to be upregulated at early time points post vaccination in salmon [116–117]. Olive flounder injected with *E*. *tarda* derived vesicles has been reported to display immunogenic alterations due to MV exposure, including upregulation of *il-1β* and *il-6* detected in kidney samples at 3 hours post challenge and maintained up to 5 days [38]. Similar results are found for Japanese flounder immunized with vesicles isolated from *Vibrio anguillarum*, where they observed, an increased production of pro-inflammatory cytokines, including *tnfα*, *il-1β* and *il-6*, during the first 48 hours [118]. Furthermore, when MVs isolated from *E*. *tarda* was tested as a vaccine candidate towards edwardsiellosis, it provided an equal level of protection as Formalin-killed *E*. *tarda* cells [38]. An activation of the innate immune system, including upregulation of pro-inflammatory cytokines, plays an important role in the adaptive immune response by attracting the antigen presenting cells [119]. Thus, the up-regulation of immune-related genes detected in this study, might indicate a potential activation of the host’s immune system initiated by the MVs. Whether this activation is mediated by i.e. toll-like receptors (TLRs) or not, is not known. Considering the composition of MVs, containing several molecules and proteins identified as pathogen associated molecular pattern (PAMPS) including LPS, carbohydrates, heat-shock proteins (HSPs) and nuclei sequence motifs suggest the participation of TLRs as a bridge between innate and adaptive immunity, making *P*. *salmonis* MVs interesting as a vaccine candidate. As mortalities were observed for fish immunized with vesicles isolated from LF-89, further dose-response studies are needed to evaluate the vesicles long-term effect. However, as an effect was observed both *in vivo* as well as *in vitro* by the *P*. *salmonis* derived MVs, the vesicles could be an integral part of the bacterium’s pathogenesis as suggested by others [52]. It could be argued that mortalities observed in the zebrafish when exposed LF-89 derived MVs compared to the two other strains are caused by differences in the LPS. LPS isolated from *E*. *coli* has been shown to have a limited effect in adult zebrafish [120], but an immunogenic response has been observed in zebrafish larva exposed to *E*. *coli* and *P*. *aeruginosa* LPS [121–122]. There is, however, still a lack of knowledge regarding the immunogenic effect of LPS from fish pathogens, and studies of *P*. *salmonis* derived LPS would be interesting to follow up in future studies.

## Conclusion

The present study is the first investigation of MV proteomes from bacterial species isolated from a wide geographical area. It is also the first in-depth analysis of MVs from multiple *P*. *salmonis* isolates. We show that MVs derived from LF-89, a high-virulent strain isolated from Chile, differs compared to the MVs of a Norwegian and Canadian strain, isolated from low infection-associated areas. According to the number of shared proteins in the MVs, we can identify two cluster, one that include NVI 5692 and NVI 5892 derived vesicles and a second one including LF-89 MVs. In general, these results illustrate that the MVs proteome analyzed from one bacterial strain is not representative of all bacterial strains within the same species. Furthermore, our findings also demonstrate that *P*. *salmonis* derived MVs are able to associate within both primary and commercial fish cells suggesting a potential role with fish immune cells. The use of zebrafish as a model for studies of MVs allowed us to investigate toxicity and the immunogenic effects upon injection with the bacterial derived vesicles indicating that the *P*. *salmonis* derived MVs are able to modulate the host’s immune response. These data indicates that the *P*. *salmonis* derived MVs could be important for the bacterium’s virulence, which also make them relevant as potential vaccine candidate against SRS.

## Ethics Statement

All animal experiments were approved by the Norwegian Animal Research Authority, approval no. 2014/3409, FOTS ID 5959/ and 2014/239007 FOTS ID 7028 and treated according to institutional guidelines.

## Supporting Information

S1 Fig Image analysis of membrane vesicles from *Piscirickettsia salmonis*. Electron microscopy image analysis of MVs, exemplified by NVI 5692, showing the presences of both single membrane vesicles (OMV) consisting of an outer membrane (OM), and double membrane vesicles (I-OMV) containing a periplasmic membrane (PM) and an outer membrane.
(PDF)

S2 Fig Comparison of isolated membrane fractions and membrane vesicles from *Piscirickettsia salmonis*. Membrane fractions (M) and membrane vesicles (MVs) (20μg) isolated from three strains of *P*. *salmonis*, NVI 5692, NVI 5892 and LF-89 were applied to 12% SDS-PAGE and stained with coomassie blue.
(PDF)

S3 Fig Cellular localization of the theoretical proteome of LF-89. Pie chart showing the cellular localization for the theoretical proteome of *P*. *salmonis* LF-89 (ATCC VR 1361).
(PDF)

S4 Fig Cellular incorporation of membrane vesicles isolated from *Piscirickettsia salmonis*. Percentage of SHK-1, Atlantic salmon cell line, spleen and kidney primary cells isolated from adult zebrafish able to incorporate 10μg/mL of FITC conjugated membrane vesicles isolated from *P*. *salmonis* strains LF-89, NVI 5692 and NVI 5892 as assessed by flow cytometry (n = 3).
(PDF)

S1 File Liquid chromatography-mass spectrometry (LC-MS). (PDF)

S1 Table Primers used for RT-qPCR in this study. (PDF)

S2 Table Proteins identified in *Piscirickettsia salmonis* LF-89 MVs analyzed by mass spectrometry. (PDF)

S3 Table Proteins identified in *Piscirickettsia salmonis* NVI 5692 MVs analyzed by mass spectrometry. (PDF)

S4 Table Proteins identified in *Piscirickettsia salmonis* NVI 5892 MVs analyzed by mass spectrometry. (PDF)
