# Supplementary material for: Comparative Analysis of Membrane Vesicles from Three Piscirickettsia salmonis Isolates Reveals Differences in Vesicle Characteristics
Source: PLoS One. 2016 Oct 20;11(10):e0165099. doi: 10.1371/journal.pone.0165099 (PMC5072724; doi:10.1371/journal.pone.0165099)
Supplement: S1 Fig — Electron microscopy image analysis of MVs, exemplified by NVI 5692, showing the presences of both single membrane vesicles (OMV) consisting of an outer membrane (OM), and double membrane vesicles (I-OMV) containing a periplasmic membrane (PM) and an outer membrane. (PDF) [file pone.0165099.s001.pdf]

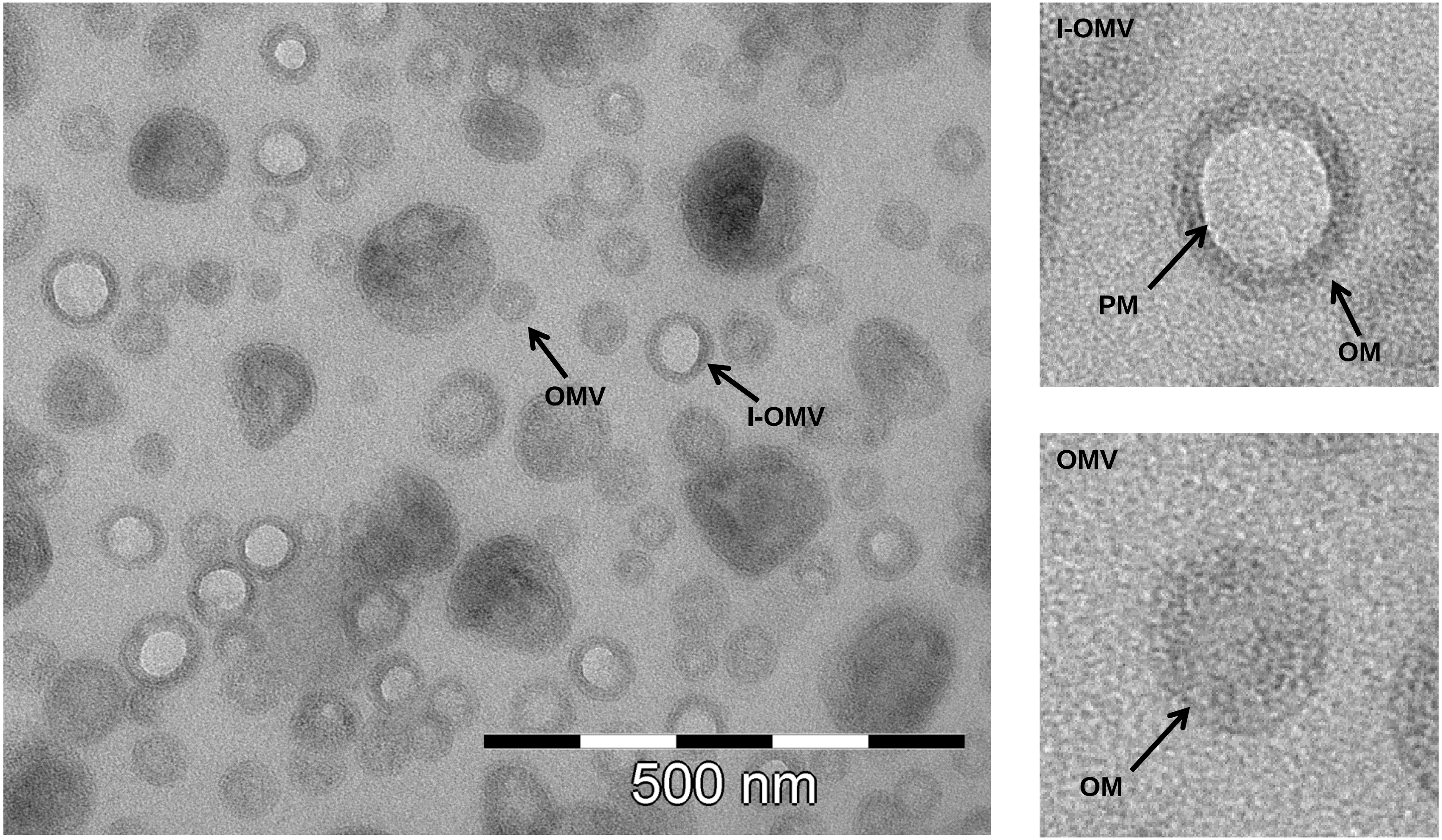

S1 Fig. Image analysis of membrane vesicles from *Piscirickettsia salmonis*. Electron microscopy image analysis, exemplified by NVI 5692, showing the presences of both single membrane vesicles (OMV) consisting of an outer membrane (OM), and double membrane vesicles (I-OMV) containing a periplasmic membrane (PM) and an outer membrane.
