# Supplementary material for: Comparative Analysis of Membrane Vesicles from Three Piscirickettsia salmonis Isolates Reveals Differences in Vesicle Characteristics
Source: PLoS One. 2016 Oct 20;11(10):e0165099. doi: 10.1371/journal.pone.0165099 (PMC5072724; doi:10.1371/journal.pone.0165099)
Supplement: S2 Fig — Membrane fractions (M) and membrane vesicles (MVs) (20μg) isolated from three strains of P. salmonis, NVI 5692, NVI 5892 and LF-89 were applied to 12% SDS-PAGE and stained with coomassie blue. (PDF) [file pone.0165099.s002.pdf]

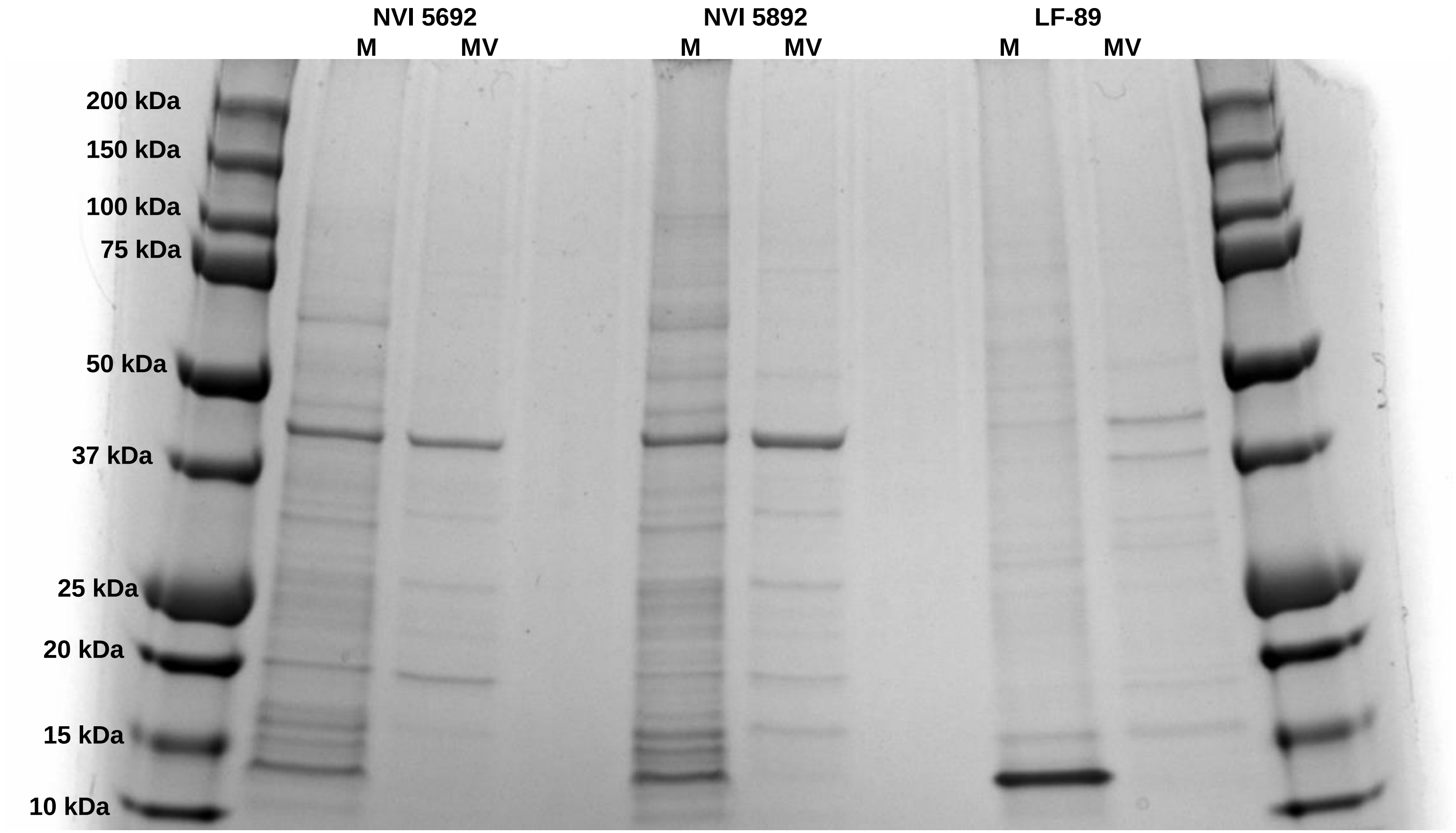

S2 Fig. Comparison of isolated membrane fraction and membrane vesicles from *Piscirickettsia salmonis*. Membrane fractions (M) and membrane vesicles (MV) (20 µg) isolated from three strains of *P. salmonis*, NVI 5692, NVI 5892 and LF-89 were applied to 12% SDS-PAGE and stained with coomassie blue
