# Supplementary material for: Comparative Analysis of Membrane Vesicles from Three Piscirickettsia salmonis Isolates Reveals Differences in Vesicle Characteristics
Source: PLoS One. 2016 Oct 20;11(10):e0165099. doi: 10.1371/journal.pone.0165099 (PMC5072724; doi:10.1371/journal.pone.0165099)
Supplement: S3 Fig — Pie chart showing the cellular localization for the theoretical proteome of P. salmonis LF-89 (ATCC VR 1361). (PDF) [file pone.0165099.s003.pdf]

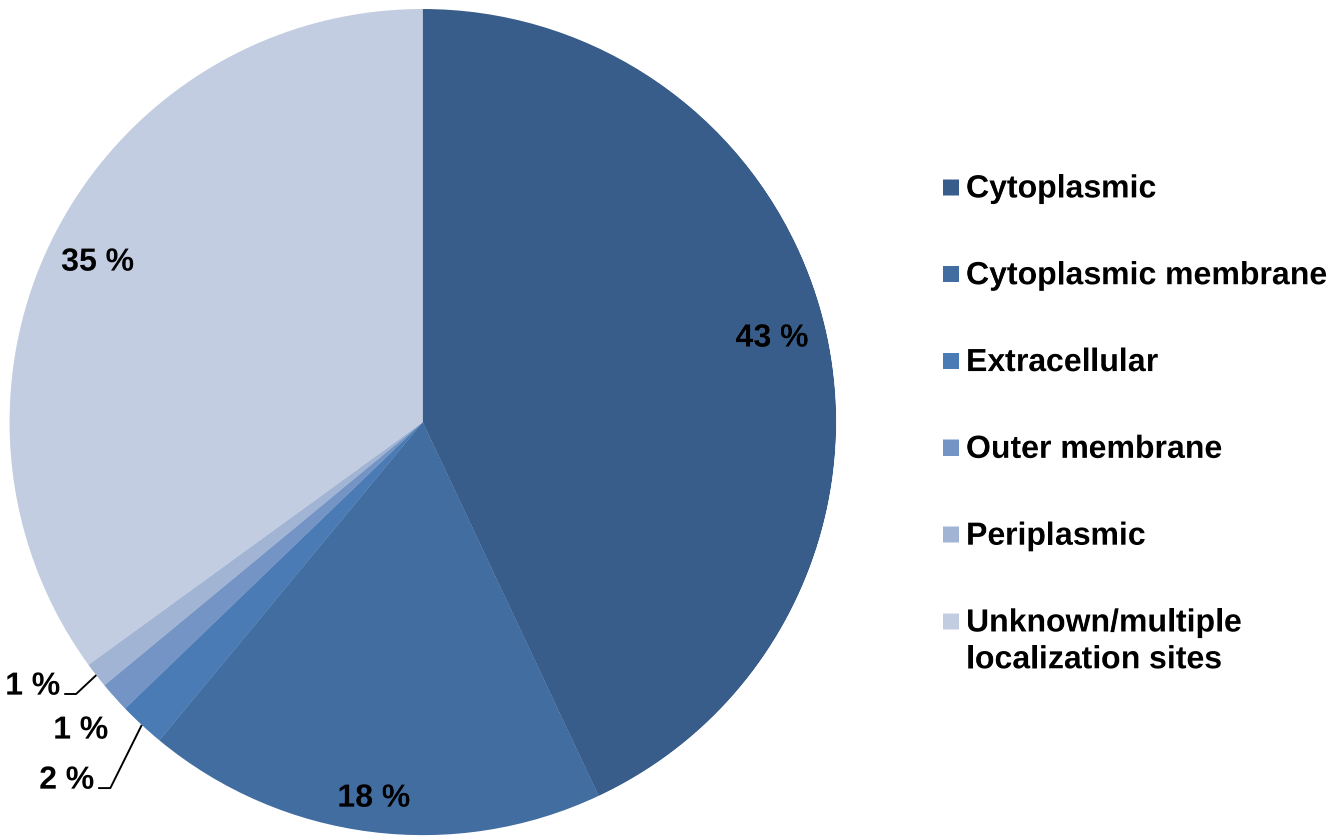

S3 Fig. Cellular localization of the theoretical proteome of LF-89. Pie chart showing the cellular localization for the theoretical proteome of *P. salmonis* LF-89 (ATCC VR 1361).
