# Supplementary material for: Comparative Analysis of Membrane Vesicles from Three Piscirickettsia salmonis Isolates Reveals Differences in Vesicle Characteristics
Source: PLoS One. 2016 Oct 20;11(10):e0165099. doi: 10.1371/journal.pone.0165099 (PMC5072724; doi:10.1371/journal.pone.0165099)
Supplement: S4 Fig — Percentage of SHK-1, Atlantic salmon cell line, spleen and kidney primary cells isolated from adult zebrafish able to incorporate 10μg/mL of FITC conjugated membrane vesicles isolated from P. salmonis strains LF-89, NVI 5692 and NVI 5892 as assessed by flow cytometry (n = 3). (PDF) [file pone.0165099.s004.pdf]

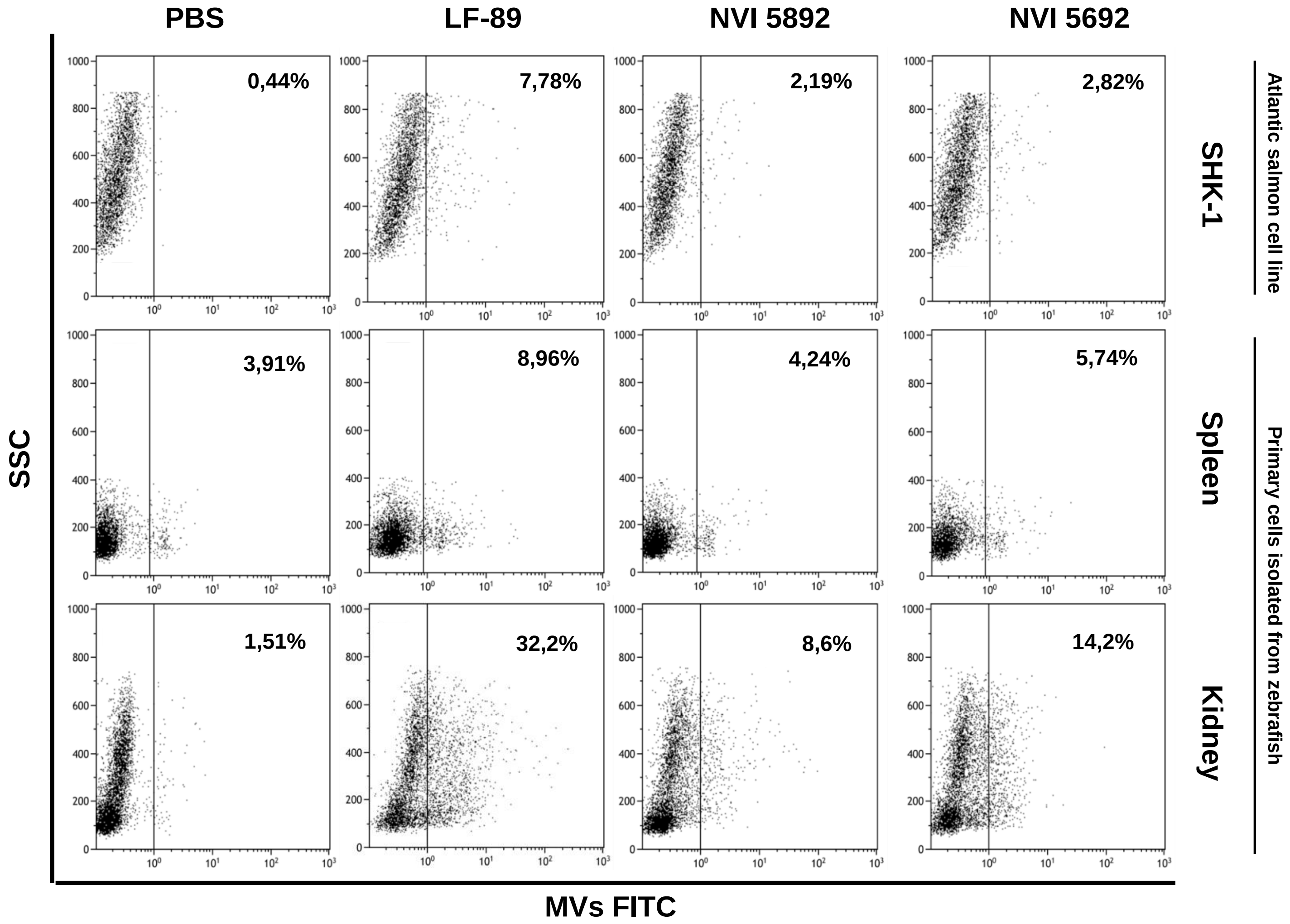

S4 Fig. Cellular incorporation of membrane vesicles isolated from *Piscirickettsia salmonis*. Percentage of of SHK-1, Atlantic salmon cell line, spleen and kidney primary cells isolated from adult zebrafish able to incorporate 10μg/mL of FITC conjugated membrane vesicles isolated from *P. salmonis* strains LF-89, NVI 5692 and NVI 5892 as assessed by flow cytometry (n=3).
