# Supplementary material for: Comparative Analysis of Membrane Vesicles from Three Piscirickettsia salmonis Isolates Reveals Differences in Vesicle Characteristics
Source: PLoS One. 2016 Oct 20;11(10):e0165099. doi: 10.1371/journal.pone.0165099 (PMC5072724; doi:10.1371/journal.pone.0165099)
Supplement: S1 File — (PDF) [file pone.0165099.s005.pdf]

S1 File. Liquid chromatography-mass spectrometry (LC-MS)

The tryptic peptides were analyzed using an Ultimate 3000 nano-UHPLC system connected to a Q Exactive mass spectrometer (Thermo Fisher Scientific, Bremen, Germany) equipped with a nano electrospray ion source. For liquid chromatography separation, an Acclaim PepMap 100 column (C18, 3 µm beads, 100 Å, 75 µm inner diameter, 50 cm length) (Dionex, Sunnyvale CA, USA) was used. A flow rate of 300 nL/min was employed with a solvent gradient of 4-35% B in 207 min, to 50% B in 20 min and then to 80% B in 2 min. Solvent A was 0.1% formic acid and solvent B was 0.1% formic acid/90% acetonitrile.

The mass spectrometer was operated in the data-dependent mode to automatically switch between MS and MS/MS acquisition. Survey full scan MS spectra (from m/z 300 to 2,000) were acquired with the resolution R = 70,000 at m/z 200, after accumulation to a target of 1e6. The maximum allowed ion accumulation times were 60 ms. The method used allowed sequential isolation of up to the ten most intense ions, depending on signal intensity (intensity threshold 1.7e4), for fragmentation using higher collision induced dissociation (HCD) at a target value of 10,000 charges and a resolution R = 17,500 Target ions already selected for MS/MS were dynamically excluded for 60 sec. The isolation window was m/z = 2 without offset. The maximum allowed ion accumulation for the MS/MS spectrum was 60 ms. For accurate mass measurements, the lock mass option was enabled in MS mode.
