# Supplementary material for: Comparative Analysis of Membrane Vesicles from Three Piscirickettsia salmonis Isolates Reveals Differences in Vesicle Characteristics
Source: PLoS One. 2016 Oct 20;11(10):e0165099. doi: 10.1371/journal.pone.0165099 (PMC5072724; doi:10.1371/journal.pone.0165099)
Supplement: S1 Table — (PDF) [file pone.0165099.s006.pdf]

S1 Table. Primers used for RT-qPCR in this study

Gene name	Gene symbol	Forward primer	Reverse primer	Product size	Accession number	Reference
Eukaryotic translation elongation factor 1 alpha 1, like 1	*eef1α11 1*	CTT CTC AGG CTG ACT GTG C	CCG CTA GCA TTA CCC TCC	358 bp	NM_131263.1	[1]
18S rRNA	*zgc:158 463*	GCC TGC GGC TTA ATT TGA CT	ACC ACC CAC AGA ATC GAG AAA	98 bp	NM_001098396	[2]
interleukin 1, beta	*il-1β*	QuantiTect Primer Assay Dr_il1b_1_SG		111 bp	NM_212844	QIAGEN Cat # QT02063565
interleukin 6	*il-6*	TCA ACT TCT CCA GCG TGA TG	TCT TTC CCT CTT TTC CTC CTG	73 bp	NM_001114318	[3]
interleukin 8	*il-8*	QuantiTect Primer Assay Dr_il8_1_SG		147 bp	XM_001342570	QIAGEN Cat # QT02108190
interleukin 10	*il-10*	QuantiTect Primer Assay Dr_il10_1_SG		144 bp	NM_001020785	QIAGEN Cat # QT02063922
interleukin 12a	*il-12a*	QuantiTect Primer Assay Dr_il12a_1_SG		94 bp	NM_001007107	QIAGEN Cat # QT02085300
tumor necrosis factor a	*tnfa*	QuantiTect Primer Assay Dr_tnfa_1_SG		81 bp	NM_212859	QIAGEN Cat # QT02097655
interferon, gamma 1-2	*ifng1-2*	QuantiTect Primer Assay Dr_ifng1-2_1_SG		89 bp	NM_212864	QIAGEN Cat # QT02064328
suppressor of cytokine signaling 3b	*socs3b*	QuantiTect Primer Assay Dr_socs3b_1_SG		123 bp	NM_213304	QIAGEN Cat # QT02068724
suppressor of cytokine signaling 3a	*socs3a*	QuantiTect Primer Assay Dr_socs3a_1_SG		97 bp	NM_199950	QIAGEN Cat # QT02056488
macrophage expressed 1	*mpeg1*	TAC AGC ACG GGT TCA AGT CCG T	ACT TGT GAT GAC ATG GGT GCC G	187 bp	NM_212737	[4]
TNF receptor superfamily member 5	*cd40*	AGA GTT GCC GTT AAA GGT TC	TTC TCC GTA CTC ACA TTT GG	123 bp	NM_001145246	[5]
major histocompatibility complex class II	*MHC II*	TGA CTC AAC TGT CCG TGA TA	CCA TTA GCC ATC TCC ATA GTG	195 bp	NM_001005943	[6]
Nucleotide binding and oligomerization domain 1	*nod1*	QuantiTect Primer Assay Dr_nod2_1_SG		64 bp	XM_692832	QIAGEN Cat # QT02209270
Nucleotide binding and oligomerization domain 2	*nod2*	QuantiTect Primer Assay Dr_LOC570770_1_SG		99 bp	XM_694287	QIAGEN Cat # QT02219875

References

1. Chen, J., et al., Loss of function of def selectively up-regulates Delta113p53 expression to arrest expansion growth of digestive organs in zebrafish. *Genes & Development*, **2005**. 19(23): p. 2900-11.
2. Dios, S., et al., Effect of the temperature during antiviral immune response ontogeny in teleosts. *Fish & Shellfish Immunology* , **2010.** 29(6): p. 1019-27.
3. Varela, M., et al., Characterisation, expression and ontogeny of interleukin-6 and its receptors in zebrafish (*Danio rerio*). *Developmental and Comparative Immunology,* **2012**. 37(1): p. 97-106.
4. Liu, X., et al., Notable mucosal immune responses induced in the intestine of zebrafish (*Danio rerio*) bath-vaccinated with a live attenuated *Vibrio anguillarum* vaccine. *Fish and Shellfish Immunology*, **2014**. 40(1): p. 99-108.
5. Page, D.M., et al., An evolutionarily conserved program of B-cell development and activation in zebrafish. *Blood*, **2013**. 122(8): p. e1-11.
6. Zhang, Z., et al., Immune responses of zebrafish (*Danio rerio*) induced by bath-vaccination with a live attenuated *Vibrio anguillarum* vaccine candidate. *Fish and Shellfish Immunology,* **2012**. 33(1): p. 36-41.
