# Supplementary material for: Comparative Analysis of Membrane Vesicles from Three Piscirickettsia salmonis Isolates Reveals Differences in Vesicle Characteristics
Source: PLoS One. 2016 Oct 20;11(10):e0165099. doi: 10.1371/journal.pone.0165099 (PMC5072724; doi:10.1371/journal.pone.0165099)
Supplement: S2 Table — (PDF) [file pone.0165099.s007.pdf]

Table S2. Proteins identified in *Piscirickettsia salmonis* LF-89 MVs analyzed by mass spectrometry

Proteins Identified in *Piscirickettsia salmonis* strain LF-89 MVs					
**Protein**	**Total number of spectra**	**Protein product**	**Gene locus**	**Predicted subcellular location**	**Putative function**
Outer membrane family protein	81	ERL63478.1	K661_00143	Unknown	Unknown
DNA-directed RNA polymerase subunit beta	79	ERL63118.1	K661_00502	Cytoplasmic	Translation/ transcription
Bacterial DNA-binding family protein	72	ERL63373.1	K661_00235	Unknown	Translation/ transcription
Chaperone protein DnaK	63	ERL62498.1	K661_01147	Cytoplasmic	Protein folding
60kDa chaperonin GroEL	58	ERL63008.1	K661_00639	Cytoplasmic	Protein folding
30s ribosomal protein S1	44	ERL61835.1	K661_01816	Cytoplasmic	Translation/ transcription
SH3 domain of the SH3b1 type family protein	44	ERL63011.1	K661_00616	Cytoplasmic membrane	Unknown
ATP synthase subunit beta	40	ERL63527.1	K661_00079	Cytoplasmic	Enzymatic activity
Succinyl-CoA synthetase subunit beta	37	ERL62630.1	K661_00993	Cytoplasmic	Enzymatic activity
Adenylosuccinate synthetase	35	ERL63023.1	K661_00605	Cytoplasmic	Catalytic activity
50S ribosomal protein L2	35	ERL63107.1	K661_00512	Cytoplasmic	Translation/ transcription
Pyruvate dehydrogenase E1 component	35	ERL63007.1	K661_00645	Cytoplasmic	Enzymatic activity
Translation elongation factor Tu	32	ERL63123.1	K661_00507	Cytoplasmic	Translation/ transcription
ATP synthase subunit alpha	31	ERL63511.1	K661_00081	Cytoplasmic	Transporter activity
30S ribosomal protein S10	31	ERL63124.1	K661_00508	Cytoplasmic	Translation/ transcription
Acetyl-CoA carboxylase, biotin carboxylase subunit	30	ERL62995.1	K661_00634	Cytoplasmic	Biotin carboxylase activity
GTP-binding protein TypA/BipA	30	ERL62117.1	K661_01542	Cytoplasmic membrane	GTPase activity
Adenylosuccinate lyase	30	ERL61210.1	K661_02453	Cytoplasmic	Catalytic activity
Putative uncharacterized protein	29	ERL61815.1	K661_01832	Extracellular	Unknown
Glutamine synthetase	28	ERL62820.1	K661_00817	Cytoplasmic	Catalytic activity
Aspartate--tRNA ligase	27	ERL61424.1	K661_02242	Cytoplasmic	Ligase activity
Type I secretion outer membrane , TolC family protein	27	ERL62298.1	K661_01343	Outer membrane	transporter activity
Ribonucleoside-diphosphate reductase	26	ERL62861.1	K661_00776	Cytoplasmic	Catalytic activity
Sulfate transporter family protein	26	ERL60960.1	K661_02719	Cytoplasmic membrane	Transporter activity
SurA N-terminal domain protein	26	ERL62410.1	K661_01228	Periplasmic	Protein folding

30S ribosomal protein S7	25	ERL63096.1	K661_00505	Cytoplasmic	Translation/ transcription
Outer membrane protein assembly factor BamB	25	ERL63452.1	K661_00202	Outer membrane	Outer membrane assembly
PLD-like domain protein	24	ERL62649.1	K661_00979	Unknown	Catalytic activity
Dihydrolipoyllysine-residue succinyltransferase, E2 component of oxoglutarate dehydrogenase	24	ERL62645.1	K661_00994	Cytoplasmic	Tricarboxylic acid cycle
Acetyl-coenzyme A carboxylase carboxyl transferase subunit alpha	24	ERL63463.1	K661_00136	Cytoplasmic	Enzymatic activity
DNA translocase ftsK	24	ERL61259.1	K661_02403	Cytoplasmic membrane	Translation/ transcription
Tyrosine kinase family protein	23	ERL62174.1	K661_01480	Cytoplasmic	Protein kinase activity
Lipopolysaccharide transport periplasmic protein LptA	23	ERL63347.1	K661_00276	Unknown/ multiple localization sites	Transporter activity
VacJ like lipofamily protein	23	ERL63561.1	K661_00057	Unknown/ multiple localization sites	Unknown
GTPase Era	23	ERL63402.1	K661_00257	Cytoplasmic membrane	GTPase activity
Ribonuclease E	22	ERL63435.1	K661_00190	Cytoplasmic	Enzymatic activity
Protein QmcA	22	ERL61641.1	K661_02010	Cytoplasmic	Outer membrane assembly
RNA pyrophosphohydrolase	22	ERL62690.1	K661_00954	Cytoplasmic	Ligase activity
Putative uncharacterized protein	22	ERL60989.1	K661_02693	Unknown	Unknown
Putative lipoprotein	21	ERL61739.1	K661_01913	Cytoplasmic membrane	Unknown
30S ribosomal protein S11	21	ERL63094.1	K661_00532	Cytoplasmic	Translation/ transcription
Putative uncharacterized protein	21	ERL62319.1	K661_01324	Unknown/ multiple localization sites	Unknown
tol-Pal system beta propeller repeat protein TolB	21	ERL63447.1	K661_00180	Periplasmic	Transporter activity
UvrABC system protein A/excinuclease ABC subunit A	20	ERL61138.1	K661_02532	Cytoplasmic	Catalytic activity
50S ribosomal protein L14	20	ERL63120.1	K661_00519	Cytoplasmic	Translation/ transcription
AhpC/TSA family protein	20	ERL62427.1	K661_01215	Cytoplasmic	Antioxidant activity
Peptidase Do family protein	20	ERL61942.1	K661_01712	Periplasmic	Peptidase activity
tRNA N6-adenosine threonylcarbamoyltransferase	19	ERL61798.1	K661_01858	Extracellular	Catalytic activity
50S ribosomal protein L7/L12	19	ERL63105.1	K661_00500	Unknown/ multiple localization sites	Translation/ transcription

Isocitrate dehydrogenase [NADP]	19	ERL61705.1	K661_01940	Cytoplasmic	Catalytic activity
Phosphoglycerate kinase	19	ERL62692.1	K661_00944	Cytoplasmic	Catalytic activity
Putative uncharacterized protein	18	ERL61555.1	K661_02103	Unknown/ multiple localization sites	Unknown
Cell division protein FtsZ	18	ERL63530.1	K661_00116	Cytoplasmic	GTPase activity
Glutaredoxin	18	ERL61767.1	K661_01878	Unknown/ multiple localization sites	Oxidoreductase activity
Outer membrane family protein	18	ERL62624.1	K661_01016	Unknown	Unknown
Heme ABC exporter, ATP-binding protein CcmA	18	ERL62004.1	K661_01637	Cytoplasmic	Transporter activity
30S ribosomal protein S13	17	ERL63109.1	K661_00531	Cytoplasmic	Translation/ transcription
ATP-dependent chaperone protein ClpB	17	ERL62667.1	K661_00977	Cytoplasmic	Protein folding
Thiamine-phosphate synthase	17	ERL61616.1	K661_02033	Cytoplasmic	Catalytic activity
FMN-dependent dehydrogenase family protein	17	ERL62372.1	K661_01269	Unknown/ multiple localization sites	Glutamate synthase activity
Succinyl-CoA ligase [ADP-forming] subunit alpha	17	ERL62635.1	K661_00992	Cytoplasmic	Enzymatic activity
Poly(R)-hydroxyalkanoic acid synthase, class I family protein	17	ERL62445.1	K661_01203	Cytoplasmic	Transporter activity
DNA gyrase subunit B	16	ERL63522.1	K661_00098	Cytoplasmic	Catalytic activity
tRNA modification GTPase MnmE	16	ERL63514.1	K661_00091	Cytoplasmic	Catalytic activity
DNA-directed RNA polymerase subunit alpha	16	ERL63103.1	K661_00534	Cytoplasmic	Translation/ transcription
Indole-3-glycerol phosphate synthase	16	ERL63145.1	K661_00494	Cytoplasmic	Catalytic activity
30S ribosomal protein S5	15	ERL63128.1	K661_00526	Cytoplasmic	Translation/ transcription
DNA protecting protein DprA	15	ERL62510.1	K661_01135	Unknown/ multiple localization sites	Unknown
Transcription termination factor Rho	15	ERL62818.1	K661_00824	Cytoplasmic	Translation/ transcription
Methionine--tRNA ligase	14	ERL61386.1	K661_02275	Cytoplasmic	Catalytic activity
Protein RecA	14	ERL63028.1	K661_00624	Cytoplasmic	Endonuclease activity
Delta-1-pyrroline-5-carboxylate dehydrogenase	14	ERL62434.1	K661_01213	Cytoplasmic	Dehydrogenase activity
30S ribosomal protein S18	13	ERL63390.1	K661_00228	Cytoplasmic	Translation/ transcription
ATP-dependent zinc metalloprotease FtsH	13	ERL63004.1	K661_00647	Cytoplasmic Membrane	Metallopeptidase
30S ribosomal protein S12	13	ERL63129.1	K661_00504	Cytoplasmic	Translation/ transcription

Nuclease	12	ERL60358.1	K661_03322	Cytoplasmic	Hydrolase activity
Elongation factor G	12	ERL63100.1	K661_00506	Cytoplasmic	Enzymatic activity
KamA family protein	12	ERL62198.1	K661_01449	Cytoplasmic	Catalytic activity
Translation initiation factor IF-2	12	ERL63560.1	K661_00031	Cytoplasmic	GTPase activity
Ferrous iron transport protein B	12	ERL62163.1	K661_01495	Cytoplasmic Membrane	Transporter activity
Outer membrane protein assembly factor BamE	12	ERL63567.1	K661_00069	Outer membrane	Outer membrane assembly
Macrophage killing with similarity to conjugation family protein	12	ERL62896.1	K661_00732	Unknown	Unknown
Acetyl-CoA C-acetyltransferase family protein	11	ERL63594.1	K661_00043	Cytoplasmic	Enzymatic activity
50S ribosomal protein L19	11	ERL61544.1	K661_02122	Cytoplasmic	Translation/ transcription
50S ribosomal protein L17	11	ERL63104.1	K661_00535	Cytoplasmic	Translation/ transcription
Polyribonucleotide nucleotidyltransferase	10	ERL63591.1	K661_00035	Cytoplasmic	Catalytic activity
tRNA (guanine-N(1)-)-methyltransferase	10	ERL61543.1	K661_02121	Cytoplasmic	Catalytic activity
50S ribosomal protein L22	9	ERL63119.1	K661_00514	Cytoplasmic	Translation/ transcription
DNA ligase	9	ERL62326.1	K661_01313	Cytoplasmic	Catalytic activity
CobQ/CobB/MinD/ParA nucleotide binding domain protein	9	ERL60892.1	K661_02784	Cytoplasmic	Peptidase activity
Spore coat assembly SafA domain protein	9	ERL63078.1	K661_00541	Unknown	Enzymatic activity
AAA-like domain protein	9	ERL62889.1	K661_00737	Cytoplasmic	Unknown
Glutaredoxin family protein	9	ERL62158.1	K661_01491	Cytoplasmic	Unknown
Transposase, IS4 family protein	8	ERL60903.1	K661_02769	Cytoplasmic	Transporter activity
Histidine kinase	8	ERL62934.1	K661_00699	Cytoplasmic membrane	Catalytic activity
Single-stranded DNA-binding protein	8	ERL61136.1	K661_02530	Cytoplasmic	Translation/ transcription
MutS domain II family protein	8	ERL63033.1	K661_00626	Cytoplasmic	ATP binding
Putative uncharacterized protein	7	ERL61465.1	K661_02194	Cytoplasmic	Unknown
Ribonucleoside-diphosphate reductase subunit beta	7	ERL62859.1	K661_00775	Cytoplasmic	Translation/ transcription
Transcription elongation protein nusA	7	ERL63554.1	K661_00030	Cytoplasmic	Translation/ transcription
Endonuclease/Exonuclease/ phosphatase family protein	7	ERL62544.1	K661_01095	Cytoplasmic	Endonuclease activity
Cadmium carbonic anhydrase repeat family protein	7	ERL60930.1	K661_02748	Unknown/ multiple localization sites	Unknown
Protein translocase subunit SecA	7	ERL63540.1	K661_00118	Cytoplasmic	Transporter activity
Protein translocase subunit SecD	7	ERL62918.1	K661_00714	Cytoplasmic membrane	Transporter activity
Oxoglutarate dehydrogenase (Succinyl-transferring), E1	6	ERL62637.1	K661_00995	Cytoplasmic	Enzymatic activity

component					
D-methionine-binding lipoprotein metQ	6	ERL62766.1	K661_00869	Cytoplasmic membrane	Unknown
UDP-2,3-diacylglucosamine hydrolase	6	ERL60994.1	K661_02685	Cytoplasmic membrane	Catalytic activity
ATP synthase F0, C subunit	6	ERL63516.1	K661_00084	Cytoplasmic membrane	Transporter activity
Lytic murein transglycosylase B	6	ERL62468.1	K661_01166	Cytoplasmic membrane	Transporter activity
BON domain protein	6	ERL62447.1	K661_01202	Unknown/ multiple localization sites	Unknown
Proton-translocating NADH-quinone oxidoreductase, chain L family protein	6	ERL63565.1	K661_00025	Cytoplasmic membrane	NADH dehydrogenase
Carbon storage regulator	6	ERL63016.1	K661_00621	Unknown/ multiple localization sites	Enzymatic activity
Alanine dehydrogenase	6	ERL60806.1	K661_02874	Cytoplasmic	Dehydrogenase activity
LPS-assembly protein LptD	6	ERL62408.1	K661_01227	Outer membrane	Outer membrane assembly
TonB family C-terminal domain protein	6	ERL61728.1	K661_01924	Unknown/ multiple localization sites	Transporter activity
InsA N-terminal domain protein	6	ERL60693.1	K661_02987	Unknown/ multiple localization sites	Unknown
Ribosomal-protein-alanine acetyltransferase	6	ERL62218.1	K661_01428	Cytoplasmic	Transferase activity
