# Supplementary material for: Comparative Analysis of Membrane Vesicles from Three Piscirickettsia salmonis Isolates Reveals Differences in Vesicle Characteristics
Source: PLoS One. 2016 Oct 20;11(10):e0165099. doi: 10.1371/journal.pone.0165099 (PMC5072724; doi:10.1371/journal.pone.0165099)
Supplement: S3 Table — (PDF) [file pone.0165099.s008.pdf]

S3 Table. Proteins identified in *Piscirickettsia salmonis* NVI 5692 MVs analyzed by mass spectrometry

**Proteins Identified in *Piscirickettsia salmonis* strain NVI 5692 MVs**					
**Protein**	**Total number of spectra**	**Protein product**	**Gene locus**	**Predicted subcellular location**	**Putative function**
Putative uncharacterized protein	144	ERL61815.1	K661_01832	Extracellular	Unknown
Putative uncharacterized protein	136	ERL60989.1	K661_02693	Unknown	Unknown
Prolyl oligopeptidase family protein	133	ERL60791.1	K661_02889	Unknown/ multiple localization sites	Peptidase activity
Type I secretion outer membrane, TolC family protein	89	ERL62298.1	K661_01343	Outer membrane	Transporter activity
SH3 domain of the SH3b1 type family protein	86	ERL63011.1	K661_00616	Cytoplasmic membrane	Unknown
Conjugal transfer/type IV secretion DotA/TraY family protein	81	ERL62906.1	K661_00735	Cytoplasmic membrane	Unknown
Outer membrane beta-barrel domain protein	81	ERL63261.1	K661_00363	Outer membrane	Unknown
Outer membrane protein assembly factor BamA	72	ERL63467.1	K661_00144	Outer membrane	Unknown
30s ribosomal protein S1	69	ERL61835.1	K661_01816	Cytoplasmic	Translation/ transcription
Peptidyl-prolyl cis-trans isomerase	65	ERL62123.1	K661_01513	Unknown/ multiple localization sites	Protein folding
Bacterial DNA-binding family protein	65	ERL63373.1	K661_00235	Unknown	Translation/ transcription
50S ribosomal protein L2	64	ERL63107.1	K661_00512	Cytoplasmic	Translation/ transcription
Outer membrane protein assembly factor BamD	63	ERL61949.1	K661_01702	Outer membrane	Outer membrane assembly
Chaperone protein HtpG	60	ERL63045.1	K661_00577	Cytoplasmic	Protein folding
Conjugal transfer family protein	60	ERL63157.1	K661_00475	Unknown	Transporter activity
NAD-specific glutamate dehydrogenase	59	ERL62977.1	K661_00664	Cytoplasmic	Enzymatic activity
ATP synthase subunit alpha	57	ERL63511.1	K661_00081	Cytoplasmic	Transporter activity
Chaperone protein DnaK	57	ERL62498.1	K661_01147	Cytoplasmic	Protein folding
Glycerophosphoryl diester phosphodiesterase family protein	56	ERL63010.1	K661_00612	Unknown/ multiple localization sites	Enzymatic activity
Succinyl-CoA synthetase subunit beta	50	ERL62630.1	K661_00993	Cytoplasmic	Enzymatic activity

Outer membrane protein assembly factor BamB	43	ERL63452.1	K661_00202	Outer membrane	Outer membrane assembly
50S ribosomal protein L3	40	ERL63126.1	K661_00509	Cytoplasmic	Translation/ transcription
30S ribosomal protein S3	38	ERL63101.1	K661_00515	Cytoplasmic	Translation/ transcription
Transketolase	36	ERL62694.1	K661_00946	Cytoplasmic	Enzymatic activity
30S ribosomal protein S5	36	ERL63128.1	K661_00526	Cytoplasmic	Translation/ transcription
Translation elongation factor Tu	36	ERL63123.1	K661_00507	Cytoplasmic	Translation/ transcription
Gamma-glutamyltransferase	35	ERL60775.1	K661_02905	Periplasmic	Enzymatic activity
ostA-like family protein	35	ERL62408.1	K661_01227	Outer membrane	Transporter activity
Type I secretion outer membrane, TolC family protein	35	ERL62558.1	K661_01078	Outer membrane	Transporter activity
Outer membrane family protein	35	ERL63478.1	K661_00143	Unknown	Unknown
DNA-directed RNA polymerase subunit beta	33	ERL63118.1	K661_00502	Cytoplasmic	Translation/ transcription
DSBA-like thioredoxin domain protein	33	ERL62050.1	K661_01601	Unknown	Unknown
HflK protein	32	ERL63020.1	K661_00608	Unknown	Outer membrane assembly
Pyruvate dehydrogenase E1 component	32	ERL63007.1	K661_00645	Cytoplasmic	Enzymatic activity
SurA N-terminal domain protein	32	ERL62410.1	K661_01228	Periplasmic	Protein folding
Putative dotC-like type IV secretion system protein	31	ERL62897.1	K661_00745	Unknown	Unknown
VacJ like lipofamily protein	30	ERL63561.1	K661_00057	Unknown/ multiple localization sites	Unknown
Acetyl-coenzyme A carboxylase carboxyl transferase subunit beta	30	ERL62252.1	K661_01385	Cytoplasmic	Enzymatic activity
Site-determining protein	29	ERL63059.1	K661_00595	Cytoplasmic	Enzymatic activity
Peptidase Do family protein	29	ERL61942.1	K661_01712	Periplasmic	Peptidase activity
60kDa chaperonin GroEL	29	ERL63008.1	K661_00639	Cytoplasmic	Protein folding
30S ribosomal protein S10	29	ERL63124.1	K661_00508	Cytoplasmic	Translation/ transcription
Protein QmcA	27	ERL61641.1	K661_02010	Cytoplasmic	Outer membrane assembly
DNA translocase ftsK	26	ERL61259.1	K661_02403	Cytoplasmic membrane	Translation/ transcription
30S ribosomal protein S13	26	ERL63109.1	K661_00531	Cytoplasmic	Translation/ transcription
Putative uncharacterized protein	26	ERL61555.1	K661_02103	Unknown	Unknown
Protein HflC	25	ERL63022.1	K661_00607	Unknown	Peptidase activity
Bacterial conjugation TrbI-like family protein	25	ERL62888.1	K661_00750	Unknown	Transporter activity
D-alanyl-D-alanine carboxypeptidase/D-alanyl-D-alanine-endopeptidase	25	ERL62612.1	K661_01018	Periplasmic	Enzymatic activity
Putative uncharacterized protein	24	ERL62717.1	K661_00911	Unknown	Unknown

PLD-like domain protein	24	ERL62649.1	K661_00979	Unknown	Enzymatic activity
D-alanyl-D-alanine carboxypeptidase family protein	24	ERL63219.1	K661_00410	Cytoplasmic membrane	Enzymatic activity
50S ribosomal protein L18	24	ERL63121.1	K661_00525	Cytoplasmic	Translation/ transcription
Toluene tolerance, Ttg2 family protein	24	ERL63329.1	K661_00270	Unknown	Outer membrane assembly
30S ribosomal protein S7	23	ERL63096.1	K661_00505	Cytoplasmic	Translation/ transcription
tol-Pal system beta propeller repeat protein TolB	23	ERL63447.1	K661_00180	Periplasmic	Transporter activity
Polysaccharide biosynthesis/export family protein	23	ERL63275.1	K661_00379	Unknown/ multiple localization sites	Transporter activity
Acetyl-coenzyme A carboxylase carboxyl transferase subunit alpha	23	ERL63463.1	K661_00136	Cytoplasmic	Enzymatic activity
30S ribosomal protein S12	22	ERL63129.1	K661_00504	Cytoplasmic	Translation/ Transcription
30S ribosomal protein S2	21	ERL63483.1	K661_00158	Cytoplasmic	Translation/ transcription
TPR repeat family protein	21	ERL63572.1	K661_00047	Unknown	Unknown
Putative lipoprotein	21	ERL61739.1	K661_01913	Cytoplasmic membrane	Unknown
30S ribosomal protein S11	20	ERL63094.1	K661_00532	Cytoplasmic	Translation/ transcription
Putative uncharacterized protein	19	ERL62344.1	K661_01304	Unknown	Unknown
AAA-like domain protein	18	ERL62889.1	K661_00737	Cytoplasmic	Unknown
50S ribosomal protein L16	18	ERL63097.1	K661_00516	Cytoplasmic	Translation/ transcription
Polyribonucleotide nucleotidyltransferase	18	ERL63591.1	K661_00035	Cytoplasmic	Enzymatic activity
Trypsin family protein	18	ERL63279.1	K661_00360	Periplasmic	Peptidase activity
Putative uncharacterized protein	18	ERL63074.1	K661_00568	Unknown	Unknown
30S ribosomal protein S14	18	ERL63095.1	K661_00522	Cytoplasmic	Translation/ transcription
Outer membrane protein assembly factor BamE	17	ERL63567.1	K661_00069	Outer membrane	Outer membrane assembly
30S ribosomal protein S4	17	ERL63092.1	K661_00533	Cytoplasmic	Translation/ transcription
DNA gyrase subunit A	16	ERL61836.1	K661_01813	Cytoplasmic	Enzymatic activity
TRAP transporter solute receptor, TAXI family protein	16	ERL61459.1	K661_02201	Unknown	Translation/ transcription
Thioredoxin family protein	16	ERL62966.1	K661_00669	Periplasmic	Unknown
Efflux transporter, RND family, MFP subunit	16	ERL63225.1	K661_00385	Cytoplasmic membrane	Transporter activity
Protein translocase subunit SecA	15	ERL63540.1	K661_00118	Cytoplasmic	Transporter activity
Protein translocase subunit SecD	15	ERL62918.1	K661_00714	Cytoplasmic membrane	Transporter activity
Transcription termination factor Rho	15	ERL62818.1	K661_00824	Cytoplasmic	Translation/ transcription
Oxoglutarate dehydrogenase	15	ERL62637.1	K661_00995	Cytoplasmic	Enzymatic activity

(Succinyl-transferring), E1 component					
50S ribosomal protein L25	15	ERL62243.1	K661_01399	Cytoplasmic	Translation/ transcription
Preprotein translocase, YajC subunit	15	ERL62915.1	K661_00713	Cytoplasmic membrane	Unknown
MetA-pathway of phenol degradation family protein	15	ERL62980.1	K661_00673	Unknown	Outer membrane assembly
Penicillin binding transpeptidase domain protein	14	ERL60963.1	K661_02714	Cytoplasmic membrane	Peptidase activity
DNA-directed RNA polymerase subunit alpha	14	ERL63103.1	K661_00534	Cytoplasmic	Translation/ transcription
Phosphoribosylfor-mylglycinamidine cyclo-ligase	14	ERL61959.1	K661_01687	Cytoplasmic	Enzymatic activity
Succinyl-CoA ligase [ADP-forming] subunit alpha	14	ERL62635.1	K661_00992	Cytoplasmic	Enzymatic activity
Two-component transcriptional regulatory family protein	14	ERL62969.1	K661_00656	Cytoplasmic	Translation/ transcription
30S ribosomal protein S6	14	ERL63391.1	K661_00227	Cytoplasmic	Translation/ transcription
Putative uncharacterized protein	14	ERL63263.1	K661_00353	Cytoplasmic membrane	Unknown
Cytochrome o ubiquinol oxidase, subunit I	14	ERL61528.1	K661_02130	Cytoplasmic membrane	Enzymatic activity
Mce related family protein	14	ERL63357.1	K661_00271	Unknown	Unknown
ATP-dependent protease ATPase subunit HslU	13	ERL62266.1	K661_01373	Cytoplasmic	Protein folding
AT hook motif family protein	13	ERL60509.1	K661_03171	Cytoplasmic	Translation/ transcription
Putative lipoprotein	13	ERL61947.1	K661_01699	Unknown	Unknown
Penicillin-binding protein 1B	12	ERL63553.1	K661_00046	Cytoplasmic membrane	Peptidase activity
Putative uncharacterized protein	12	ERL63239.1	K661_00396	Outer membrane	Unknown
Efflux transporter, RND family, MFP subunit	12	ERL61814.1	K661_01835	Cytoplasmic membrane	Transporter activity
Spore coat assembly SafA domain protein	12	ERL63078.1	K661_00541	Unknown	Enzymatic activity
Macrophage killing with similarity to conjugation family protein	12	ERL62896.1	K661_00732	Unknown	Unknown
Transcription elongation protein nusA	12	ERL63554.1	K661_00030	Cytoplasmic	Translation/ transcription
Glutathione synthetase/ ATP-grasp domain protein	12	ERL61291.1	K661_02372	Cytoplasmic	Enzymatic activity
Elongation factor G	12	ERL63100.1	K661_00506	Cytoplasmic	Enzymatic activity
ATP synthase subunit beta	11	ERL63527.1	K661_00079	Cytoplasmic	Enzymatic activity
SurA N-terminal domain protein	11	ERL63206.1	K661_00419	Unknown/ multiple localization sites	Protein folding
Poly(R)-hydroxyalkanoic acid synthase, class I family protein	11	ERL62445.1	K661_01203	Cytoplasmic	Transporter activity
Putative uncharacterized protein	11	ERL61212.1	K661_02455	Outer membrane	Unknown

Cell division protein FtsZ	11	ERL63530.1	K661_00116	Cytoplasmic	GTPase activity
CTP synthase	10	ERL62750.1	K661_00882	Cytoplasmic	Enzymatic activity
UvrABC system protein A/ excinuclease ABC subunit A	10	ERL61138.1	K661_02532	Cytoplasmic	Nucleotide-excision repair
50S ribosomal protein L5	10	ERL63125.1	K661_00521	Cytoplasmic	Translation/ transcription
50S ribosomal protein L4	10	ERL63099.1	K661_00510	Cytoplasmic	Translation/ transcription
Succinate dehydrogenase, flavoprotein subunit	10	ERL62641.1	K661_00997	Cytoplasmic membrane	Electron transport chain
Putative uncharacterized protein	10	ERL62076.1	K661_01573	Cytoplasmic membrane	Unknown
Colicin V production family protein	10	ERL62258.1	K661_01383	Cytoplasmic membrane	Unknown
50S ribosomal protein L6	10	ERL63130.1	K661_00524	Cytoplasmic	Translation/ transcription
DNA/RNA non-specific endonuclease family protein	10	ERL61072.1	K661_02601	Extracellular	Endonuclease activity
Membrane protein insertase YidC	10	ERL63499.1	K661_00092	Cytoplasmic membrane	Transporter activity
GTP-binding protein TypA/BipA	9	ERL62117.1	K661_01542	Cytoplasmic membrane	GTPase activity
Bacterial conjugation TrbI-like family protein	9	ERL63138.1	K661_00474	Unknown	Unknown
Protein RecA	9	ERL63028.1	K661_00624	Cytoplasmic	Endonuclease activity
Efflux transporter, RND family, MFP subunit	9	ERL62143.1	K661_01504	Cytoplasmic membrane	Transporter activity
ATP-dependent chaperone protein ClpB	9	ERL62667.1	K661_00977	Cytoplasmic	Protein folding
3-oxoacyl-[acyl-carrier-protein] synthase 2	9	ERL62439.1	K661_01194	Cytoplasmic membrane	Enzymatic activity
Lipopolysaccharide transport periplasmic protein LptA	9	ERL63347.1	K661_00276	Unknown	Transporter activity
Type IV pilus biogenesis /stability protein PilW	9	ERL63442.1	K661_00207	Outer membrane	Unknown
TrbC/VIRB2 family protein	9	ERL63150.1	K661_00479	Cytoplasmic membrane	Unknown
Signal recognition particle protein	8	ERL62778.1	K661_00877	Cytoplasmic membrane	GTPase activity
Glycine cleavage system T protein	8	ERL62954.1	K661_00680	Cytoplasmic	Enzymatic activity
NADH dehydrogenase (Quinone), G subunit	8	ERL63558.1	K661_00020	Cytoplasmic	ATP synthesis
30S ribosomal protein S9	8	ERL62157.1	K661_01492	Cytoplasmic	Translation/ transcription
Ribose-phosphate pyrophosphokinase	8	ERL62246.1	K661_01400	Cytoplasmic	Enzymatic activity
DNA-directed RNA polymerase subunit beta	8	ERL63106.1	K661_00503	Cytoplasmic	Translation/ transcription
Translation initiation factor IF-2	8	ERL63560.1	K661_00031	Cytoplasmic	GTPase activity
Putative lipoprotein	8	ERL62900.1	K661_00746	Unknown	Unknown
50S ribosomal protein L24	8	ERL63098.1	K661_00520	Cytoplasmic	Translation/ transcription
Aldehyde dehydrogenase family protein	7	ERL61002.1	K661_02673	Cytoplasmic	Oxidoreductase activity
Adenylate kinase	7	ERL61571.1	K661_02089	Cytoplasmic	Enzymatic activity

Ribonuclease E	7	ERL63435.1	K661_00190	Cytoplasmic	Enzymatic activity
YGGT family protein	7	ERL63267.1	K661_00357	Cytoplasmic membrane	Unknown
Efflux transporter, RND family, MFP subunit	7	ERL61512.1	K661_02153	Cytoplasmic membrane	Transporter activity
Dihydrolipoyllysine-residue succinyltransferase, E2 component of oxoglutarate dehydrogenase	7	ERL62645.1	K661_00994	Cytoplasmic	Tricarboxylic acid cycle
Secreted metalloprotease Mcp02	7	ERL61439.1	K661_02225	Extracellular	Peptidase activity
Putative uncharacterized protein	7	ERL61817.1	K661_01838	Unknown	Unknown
FMN-dependent dehydrogenase family protein	7	ERL62372.1	K661_01269	Unknown/ multiple localization sites	Glutamate synthase activity
Putative lipoprotein	7	ERL61323.1	K661_02342	Cytoplasmic membrane	Unknown
50S ribosomal protein L20	7	ERL62352.1	K661_01296	Cytoplasmic	Translation/ transcription
phosphopyruvate hydratase	6	ERL62738.1	K661_00884	Cytoplasmic	Enzymatic activity
Alanine dehydrogenase	6	ERL60806.1	K661_02874	Cytoplasmic	Enzymatic activity
Glutaredoxin family protein	6	ERL62259.1	K661_01390	Cytoplasmic	Electron carrier activity
Lytic murein transglycosylase B	6	ERL62468.1	K661_01166	Cytoplasmic membrane	Transporter activity
50S ribosomal protein L19	6	ERL61544.1	K661_02122	Cytoplasmic	Translation/ transcription
NADH-quinone oxidoreductase subunit C	6	ERL63551.1	K661_00016	Cytoplasmic	Transporter activity
Protein TolQ	6	ERL63421.1	K661_00183	Cytoplasmic membrane	Transporter activity
30S ribosomal protein S18	6	ERL63390.1	K661_00228	Cytoplasmic	Translation/ transcription
VirB8 family protein	6	ERL63151.1	K661_00476	Unknown	Unknown
TPR repeat family protein	6	ERL61242.1	K661_02421	Unknown	Unknown
50S ribosomal protein L21	6	ERL62152.1	K661_01484	Cytoplasmic	Translation/ transcription
Hydroxymethylglutaryl-coenzyme A reductase family protein	6	ERL63498.1	K661_00145	Cytoplasmic	Coenzyme binding
