# Supplementary material for: Comparative Analysis of Membrane Vesicles from Three Piscirickettsia salmonis Isolates Reveals Differences in Vesicle Characteristics
Source: PLoS One. 2016 Oct 20;11(10):e0165099. doi: 10.1371/journal.pone.0165099 (PMC5072724; doi:10.1371/journal.pone.0165099)
Supplement: S4 Table — (PDF) [file pone.0165099.s009.pdf]

S4 Table. Proteins identified in *Piscirickettsia salmonis* NVI 5892 MVs analyzed by mass spectrometry

**Proteins Identified in *Piscirickettsia salmonis* strain NVI 5892 MVs**					
**Protein**	**Total number of spectra**	**Protein product**	**Gene locus**	**Predicted subcellular location**	**Putative function**
Putative uncharacterized protein	171	ERL61815.1	K661_01832	Extracellular	Unknown
Peptidyl-prolyl cis-trans isomerase	140	ERL62123.1	K661_01513	Unknown/ multiple localization sites	Protein folding
Outer membrane beta-barrel domain protein	104	ERL63261.1	K661_00363	Outer membrane	Unknown
Outer membrane protein assembly factor BamA	89	ERL63467.1	K661_00144	Outer membrane	Unknown
Prolyl oligopeptidase family protein	83	ERL60791.1	K661_02889	Unknown/ multiple localization sites	Peptidase activity
Type I secretion outer membrane, TolC family protein	82	ERL62298.1	K661_01343	Outer membrane	Transporter activity
Outer membrane family protein	79	ERL63478.1	K661_00143	Unknown	Unknown
Chaperone protein DnaK	74	ERL62498.1	K661_01147	Cytoplasmic	Protein folding
SH3 domain of the SH3b1 type family protein	72	ERL63011.1	K661_00616	Cytoplasmic membrane	Unknown
Conjugal transfer family protein	71	ERL63157.1	K661_00475	Unknown	Transporter activity
OmpA family protein	68	ERL63431.1	K661_00179	Outer membrane	Porin activity
Outer membrane protein assembly factor BamD	64	ERL61949.1	K661_01702	Outer membrane	Outer membrane assembly
Bacterial DNA-binding family protein	62	ERL63373.1	K661_00235	Unknown	Translation/ transcription
Conjugal transfer/type IV secretion DotA/TraY family protein	61	ERL62906.1	K661_00735	Cytoplasmic membrane	Unknown
Glycerophosphoryl diester phosphodiesterase family protein	57	ERL63010.1	K661_00612	Unknown/ multiple localization sites	Enzymatic activity
50S ribosomal protein L2	57	ERL63107.1	K661_00512	Cytoplasmic	Translation/ transcription
30s ribosomal protein S1	55	ERL61835.1	K661_01816	Cytoplasmic	Translation/ transcription
Putative uncharacterized protein	52	ERL60989.1	K661_02693	Unknown	Unknown
ostA-like family protein	48	ERL62408.1	K661_01227	Outer membrane	Transporter activity
DSBA-like thioredoxin	46	ERL62050.1	K661_01601	Unknown	Unknown

domain protein					
ATP synthase subunit alpha	46	ERL63511.1	K661_00081	Cytoplasmic	Transporter activity
Type I secretion outer membrane, TolC family protein	45	ERL62558.1	K661_01078	Outer membrane	Transporter activity
Putative uncharacterized protein	44	ERL62717.1	K661_00911	Unknown	Unknown
Outer membrane protein assembly factor BamB	43	ERL63452.1	K661_00202	Outer membrane	Outer membrane assembly
Gamma-glutamyltransferase	43	ERL60775.1	K661_02905	Periplasmic	Enzymatic activity
SurA N-terminal domain protein	42	ERL62410.1	K661_01228	Periplasmic	Protein folding
50S ribosomal protein L3	40	ERL63126.1	K661_00509	Cytoplasmic	Translation/ transcription
Site-determining protein	39	ERL63059.1	K661_00595	Cytoplasmic	Enzymatic activity
Translation elongation factor Tu	36	ERL63123.1	K661_00507	Cytoplasmic	Translation/ transcription
Bacterial conjugation TrbI-like family protein	36	ERL62888.1	K661_00750	Unknown	Transporter activity
Peptidase Do family protein	34	ERL61942.1	K661_01712	Periplasmic	Peptidase activity
Chaperone protein HtpG	34	ERL63045.1	K661_00577	Cytoplasmic	Protein folding
Succinyl-CoA synthetase subunit beta	33	ERL62630.1	K661_00993	Cytoplasmic	Enzymatic activity
Toluene tolerance, Ttg2 family protein	30	ERL63329.1	K661_00270	Unknown	Outer membrane assembly
30S ribosomal protein S5	30	ERL63128.1	K661_00526	Cytoplasmic	Translation/ transcription
60kDa chaperonin GroEL	30	ERL63008.1	K661_00639	Cytoplasmic	Protein folding
Cadmium carbonic anhydrase repeat family protein	30	ERL60930.1	K661_02748	Unknown	Unknown
Putative uncharacterized protein	26	ERL61555.1	K661_02103	Unknown	Unknown
Efflux transporter, RND family, MFP subunit	26	ERL61814.1	K661_01835	Cytoplasmic membrane	Transporter activity
PLD-like domain protein	26	ERL62649.1	K661_00979	Unknown	Enzymatic activity
Acetyl-coenzyme A carboxylase carboxyl transferase subunit beta	25	ERL62252.1	K661_01385	Cytoplasmic	Enzymatic activity
VacJ like lipofamily protein	25	ERL63561.1	K661_00057	Unknown/ multiple localization sites	Unknown
Putative lipoprotein	24	ERL61739.1	K661_01913	Cytoplasmic membrane	Unknown
30S ribosomal protein S10	24	ERL63124.1	K661_00508	Cytoplasmic	Translation/ transcription
Protein QmcA	23	ERL61641.1	K661_02010	Cytoplasmic	Outer membrane assembly
Putative dotC-like type IV secretion system protein	22	ERL62897.1	K661_00745	Unknown	Unknown
DNA translocase ftsK	22	ERL61259.1	K661_02403	Cytoplasmic membrane	Translation/ transcription
HflK protein	21	ERL63020.1	K661_00608	Unknown	Outer membrane assembly
Phospholipase A1 family protein	21	ERL62999.1	K661_00642	Outer membrane	Phospholipase activity
SurA N-terminal domain	20	ERL63206.1	K661_00419	Unknown/	Protein folding

protein				multiple localization sites	
Transketolase	19	ERL62694.1	K661_00946	Cytoplasmic	Enzymatic activity
D-alanyl-D-alanine carboxypeptidase family protein	19	ERL63219.1	K661_00410	Cytoplasmic membrane	Enzymatic activity
D-alanyl-D-alanine carboxypeptidase/D-alanyl-D-alanine-endopeptidase	19	ERL62612.1	K661_01018	Periplasmic	Enzymatic activity
tol-Pal system beta propeller repeat protein TolB	18	ERL63447.1	K661_00180	Periplasmic	Transporter activity
Acetyl-coenzyme A carboxylase carboxyl transferase subunit alpha	18	ERL63463.1	K661_00136	Cytoplasmic	Enzymatic activity
TPR repeat family protein	18	ERL63572.1	K661_00047	Unknown	Unknown
Polysaccharide biosynthesis/export family protein	18	ERL63275.1	K661_00379	Unknown/ multiple localization sites	Transporter activity
Heme ABC exporter, ATP-binding protein CcmA	17	ERL62004.1	K661_01637	Cytoplasmic	Transporter activity
Preprotein translocase, YajC subunit	17	ERL62915.1	K661_00713	Cytoplasmic membrane	Unknown
Protein HflC	17	ERL63022.1	K661_00607	Unknown	Peptidase activity
30S ribosomal protein S4	16	ERL63092.1	K661_00533	Cytoplasmic	Translation/ transcription
30S ribosomal protein S2	16	ERL63483.1	K661_00158	Cytoplasmic	Translation/ transcription
30S ribosomal protein S11	15	ERL63094.1	K661_00532	Cytoplasmic	Translation/ transcription
30S ribosomal protein S3	15	ERL63101.1	K661_00515	Cytoplasmic	Translation/ transcription
30S ribosomal protein S12	15	ERL63129.1	K661_00504	Cytoplasmic	Translation/ transcription
Polyribonucleotide nucleotidyltransferase	14	ERL63591.1	K661_00035	Cytoplasmic	Enzymatic activity
30S ribosomal protein S6	14	ERL63391.1	K661_00227	Cytoplasmic	Translation/ transcription
Spore coat assembly SafA domain protein	14	ERL63078.1	K661_00541	Unknown	Enzymatic activity
30S ribosomal protein S7	14	ERL63096.1	K661_00505	Cytoplasmic	Translation/ transcription
30S ribosomal protein S14	14	ERL63095.1	K661_00522	Cytoplasmic	Translation/ transcription
30S ribosomal protein S13	14	ERL63109.1	K661_00531	Cytoplasmic	Translation/ transcription
Putative lipoprotein	14	ERL62971.1	K661_00658	Unknown	Unknown
Cytochrome o ubiquinol oxidase, subunit I	14	ERL61528.1	K661_02130	Cytoplasmic membrane	Enzymatic activity
Thioredoxin family protein	13	ERL62966.1	K661_00669	Periplasmic	Unknown
50S ribosomal protein L16	13	ERL63097.1	K661_00516	Cytoplasmic	Translation/ transcription
Membrane protein insertase YidC	13	ERL63499.1	K661_00092	Cytoplasmic membrane	Transporter activity
Succinate dehydrogenase, flavoprotein subunit	12	ERL62641.1	K661_00997	Cytoplasmic membrane	Electron transport chain

50S ribosomal protein L9	12	ERL63394.1	K661_00229	Cytoplasmic	Translation/ transcription
Putative lipoprotein	12	ERL61947.1	K661_01699	Unknown	Unknown
Efflux transporter, RND family, MFP subunit	12	ERL63225.1	K661_00385	Cytoplasmic membrane	Transporter activity
MMPL family protein	12	ERL61816.1	K661_01834	Cytoplasmic membrane	Transporter activity
Putative uncharacterized protein	11	ERL61817.1	K661_01838	Unknown	Unknown
Bacterial conjugation TrbI-like family protein	11	ERL63138.1	K661_00474	Unknown	Unknown
Putative uncharacterized protein	11	ERL63074.1	K661_00568	Unknown	Unknown
50S ribosomal protein L4	11	ERL63099.1	K661_00510	Cytoplasmic	Translation/ transcription
50S ribosomal protein L5	11	ERL63125.1	K661_00521	Cytoplasmic	Translation/ transcription
50S ribosomal protein L24	11	ERL63098.1	K661_00520	Cytoplasmic	Translation/ transcription
Dihydrodipicolinate synthase	11	ERL61513.1	K661_02147	Cytoplasmic	Enzymatic activity
Outer membrane protein assembly factor BamE	11	ERL63567.1	K661_00069	Outer membrane	Outer membrane assembly
Macrophage killing with similarity to conjugation family protein	10	ERL62896.1	K661_00732	Unknown	Unknown
Acetyl-CoA C-acetyltransferase family protein	10	ERL63594.1	K661_00043	Cytoplasmic	Enzymatic activity
50S ribosomal protein L7/L12	10	ERL63105.1	K661_00500	Unknown/ multiple localization sites	Translation/ transcription
Poly(R)-hydroxyalkanoic acid synthase, class I family protein	10	ERL62445.1	K661_01203	Cytoplasmic	Transporter activity
NAD-specific glutamate dehydrogenase	10	ERL62977.1	K661_00664	Cytoplasmic	Enzymatic activity
MetA-pathway of phenol degradation family protein	10	ERL62980.1	K661_00673	Unknown	Outer membrane assembly
ATP synthase subunit beta	10	ERL63527.1	K661_00079	Cytoplasmic	Enzymatic activity
Succinate dehydrogenase and fumarate reductase iron-sulfur family protein	9	ERL62631.1	K661_00996	Cytoplasmic membrane	Enzymatic activity
50S ribosomal protein L18	9	ERL63121.1	K661_00525	Cytoplasmic	Translation/ transcription
Outer membrane family protein	9	ERL62624.1	K661_01016	Unknown	Unknown
Succinyl-CoA ligase [ADP-forming] subunit alpha	9	ERL62635.1	K661_00992	Cytoplasmic	Enzymatic activity
Transcription termination factor Rho	9	ERL62818.1	K661_00824	Cytoplasmic	Translation/ transcription
Elongation factor G	8	ERL63100.1	K661_00506	Cytoplasmic	Enzymatic activity
AAA-like domain protein	8	ERL62889.1	K661_00737	Cytoplasmic	Unknown
DNA/RNA non-specific endonuclease family protein	8	ERL61072.1	K661_02601	Extracellular	Endonuclease activity
Protein translocase subunit	8	ERL63540.1	K661_00118	Cytoplasmic	Transporter

SecA					activity
Putative uncharacterized protein	8	ERL63263.1	K661_00353	Cytoplasmic membrane	Unknown
FMN-dependent dehydrogenase family protein	8	ERL62372.1	K661_01269	Unknown/ multiple localization sites	Glutamate synthase activity
DNA-directed RNA polymerase subunit alpha	8	ERL63103.1	K661_00534	Cytoplasmic	Translation/ transcription
Protein translocase subunit SecD	7	ERL62918.1	K661_00714	Cytoplasmic membrane	Transporter activity
Thioredoxin	7	ERL62824.1	K661_00823	Cytoplasmic	Oxidoreductase activity
TRAP transporter solute receptor, TAXI family protein	7	ERL61459.1	K661_02201	Unknown	Translation/ transcription
Ribose-phosphate pyrophosphokinase	7	ERL62246.1	K661_01400	Cytoplasmic	Enzymatic activity
Lipopolysaccharide transport periplasmic protein LptA	7	ERL63347.1	K661_00276	Unknown	Transporter activity
YGGT family protein	7	ERL63267.1	K661_00357	Cytoplasmic membrane	Unknown
Dihydrolipoyllysine-residue succinyltransferase, E2 component of oxoglutarate dehydrogenase	7	ERL62645.1	K661_00994	Cytoplasmic	Tricarboxylic acid cycle
AT hook motif family protein	7	ERL60509.1	K661_03171	Cytoplasmic	Unknown
Mce related family protein	7	ERL63357.1	K661_00271	Unknown	Unknown
50S ribosomal protein L25	7	ERL62243.1	K661_01399	Cytoplasmic	Translation/ transcription
Putative lipoprotein	7	ERL61463.1	K661_02203	Unknown	Unknown
Putative lipoprotein	7	ERL62900.1	K661_00746	Unknown	Unknown
30S ribosomal protein S18	6	ERL63390.1	K661_00228	Cytoplasmic	Translation/ transcription
VirB8 family protein	6	ERL63151.1	K661_00476	Unknown	Unknown
30S ribosomal protein S19	6	ERL63122.1	K661_00513	Cytoplasmic	Translation/ transcription
50S ribosomal protein L28	6	ERL62116.1	K661_01537	Cytoplasmic	Translation/ transcription
ATP synthase F0, B subunit	6	ERL63538.1	K661_00083	Cytoplasmic membrane	Enzymatic activity
Superoxide dismutase [Cu-Zn]	6	ERL60975.1	K661_02704	Periplasmic	Enzymatic activity
Protein TolQ	6	ERL63421.1	K661_00183	Cytoplasmic membrane	Transporter activity
50S ribosomal protein L15	6	ERL63112.1	K661_00528	Cytoplasmic	Translation/ transcription
Alanine dehydrogenase	6	ERL60806.1	K661_02874	Cytoplasmic	Enzymatic activity
Penicillin-binding protein 1B	6	ERL63553.1	K661_00046	Cytoplasmic membrane	Peptidase activity
Putative lipoprotein	6	ERL61517.1	K661_02148	Unknown	Unknown
DNA-directed RNA polymerase subunit beta	6	ERL63106.1	K661_00503	Cytoplasmic	Translation/ transcription
Lytic murein transglycosylase B	6	ERL62468.1	K661_01166	Cytoplasmic membrane	Transporter activity
BON domain protein	6	ERL62447.1	K661_01202	Unknown/ multiple localization sites	Unknown
Carbon storage regulator	6	ERL63016.1	K661_00621	Unknown/	Enzymatic activity

				multiple localization sites	
AhpC/TSA family protein	6	ERL62427.1	K661_01215	Cytoplasmic	Antioxidant activity
Cell division protein FtsZ	6	ERL63530.1	K661_00116	Cytoplasmic	GTPase activity
DNA-directed RNA polymerase subunit beta	6	ERL63118.1	K661_00502	Cytoplasmic	Translation/ transcription
Signal recognition particle protein	6	ERL62778.1	K661_00877	Cytoplasmic membrane	GTPase activity
ATP-dependent Clp protease ATP-binding subunit ClpX	6	ERL63217.1	K661_00422	Cytoplasmic	Peptidase activity
Tol-pal system protein YbgF	6	ERL63436.1	K661_00177	Unknown	Unknown
Phosphoribosylfor-mylglycinamidine cyclo-ligase	6	ERL61959.1	K661_01687	Cytoplasmic	Enzymatic activity
